# Intercellular Adhesion Molecule-1-Induced Posttraumatic Brain Injury Neuropathology in the Prefrontal Cortex and Hippocampus Leads to Sensorimotor Function Deficits and Psychological Stress

**DOI:** 10.1523/ENEURO.0242-21.2021

**Published:** 2021-07-06

**Authors:** Saurav Bhowmick, Anitha Malat, Danielle Caruso, Nizmi Ponery, Veera D’Mello, Christina Finn, P. M. Abdul-Muneer

**Affiliations:** 1 Laboratory of CNS injury and Molecular Therapy, JFK Neuroscience Institute, Hackensack Meridian Health JFK University Medical Center, Edison, NJ 08820; 2 Department of Neurology, Hackensack Meridian School of Medicine, Nutley, NJ 07110

**Keywords:** traumatic brain injury, ICAM-1 signaling, LFA-1 and Mac-1, cerebral blood flow, neuroinflammation, neurodegeneration

## Abstract

Intercellular adhesion molecule-1 (ICAM-1) promotes adhesion and transmigration of circulating leukocytes across the blood-brain barrier (BBB). Traumatic brain injury (TBI) causes transmigrated immunocompetent cells to release mediators [function-associated antigen (LFA)-1 and macrophage-1 antigen (Mac-1)] that stimulate glial and endothelial cells to express ICAM-1 and release cytokines, sustaining neuroinflammation and neurodegeneration. Although a strong correlation exists between TBI-mediated inflammation and impairment in functional outcome following brain trauma, the role of ICAM-1 in impairing functional outcome by inducing neuroinflammation and neurodegeneration after TBI remains inconclusive. The experimental TBI was induced *in vivo* by fluid percussion injury (FPI; 10 and 20 psi) in wild-type (WT) and *ICAM-1*^−/−^ mice and *in vitro* by stretch injury (3 psi) in brain endothelial cells. We manipulate ICAM-1 pharmacologically and genetically and conducted several biochemical analyses to gain insight into the mechanisms underlying ICAM-1-mediated neuroinflammation and performed rotarod, grid-walk, sucrose preference, and light-dark tests to assess functional outcome. TBI-induced ICAM-1-mediated neuroinflammation and cell death occur via LFA-1 or Mac-1 signaling pathways that rely on oxidative stress, matrix metalloproteinase (MMP), and vascular endothelial growth factor (VEGF) pathways. The deletion or blocking of ICAM-1 resulted in a better outcome in attenuating neuroinflammation and cell death as marked by the markers such as NF-kB, IL-1β, TNF-α, cleaved-caspase-3 (cl-caspase-3), Annexin V, and by terminal deoxynucleotidyl transferase dUTP nick end labeling (TUNEL), and Trypan blue staining. ICAM-1 deletion in TBI improves sensorimotor, depression, and anxiety-like behavior with significant upregulation of norepinephrine (NE), dopamine (DA) D1 receptor (DAD1R), serotonin (5-HT)1AR, and neuropeptide Y (NPY). This study could establish the significance of ICAM-1 as a novel therapeutic target against the pathophysiology to establish functional recovery after TBI.

## Significance Statement

Intercellular adhesion molecule-1 (ICAM-1) is known to initiate neuroinflammatory responses and causes neurodegeneration and cognitive and sensory-motor deficits in several pathophysiological conditions. However, the mechanisms of ICAM-1-mediated neuroinflammation and cell death, and its link with functional deficits following traumatic brain injury (TBI) are not clear. In this study, using an *in vitro* stretch injury in human brain microvascular endothelial cells (hBMVECs) and an animal model of fluid percussion injury (FPI), we elucidated the mechanisms of activation of ICAM-1 signaling and subsequent neuroinflammation and neurodegeneration leading to sensorimotor deficits and psychological stress. We propose ICAM-1 signaling cascade as a target for developing new therapeutic strategies against TBI related neurologic diseases.

## Introduction

One of the major hallmarks of early events of traumatic brain injury (TBI) is neuroinflammation that stimulates secondary cell death (Frank and Lisanti, 2008; Bhowmick et al., 2018). Strong evidence in different models of animal focal brain injury suggests a link between the accumulation of leukocytes within the brain to increased blood-brain barrier (BBB) permeability and adhesion molecule expression (Daneman and Prat, 2015; Bhowmick et al., 2019b). Leukocytes-mediated neuroinflammation is conducted via a cascade of molecular steps wherein it involves recognition of binding sites by leukocytes and tight adherence of leukocytes rolling on the endothelium (Bevilacqua, 1993; Sumagin et al., 2011). Intercellular adhesion molecule-1 (ICAM-1) is a transmembrane glycoprotein of the Ig-superfamily constitutively expresses at low levels in the CNS (Dietrich, 2002); however, it is abundantly present during certain neuropathophysiology conditions (Sobel et al., 1990). ICAM-1 on the vascular endothelium can serve as a ligand for both leukocyte function-associated antigen (LFA)-1, a receptor found on leukocytes, and macrophage-1 antigen (Mac-1), a receptor found on neutrophils, monocytes, and macrophages (Ding et al., 1999; Henderson et al., 2001). The binding of ICAM-1 to its integrin counterparts LFA-1 and Mac-1 mediates leukocyte adhesion, increases vascular permeability and loss of the endothelial barrier, and rearrangement of the actin cytoskeleton (Sumagin et al., 2011).

Although in recent years, the vascular pathology in the brain has been studied extensively, the pathogenic mechanisms that contribute to disease progression remain elusive. Our recent studies indicate that inflammatory response is modulated by oxidative stress in TBI (Abdul-Muneer et al., 2013, 2015, 2018) and leads to disruption of BBB, and activates inflammatory signaling (Abdul-Muneer et al., 2017b; Patel et al., 2017). Oxidative stress alters the signaling pathways that regulate the immune system (McKee and Lukens, 2016) and causes the infiltration of CD4^+^T cells into the CNS during the neuroinflammatory and neurodegenerative processes (González and Pacheco, 2014). TBI-mediated neuroinflammation also produces a wide-ranging deficit in sensorimotor and cognitive functions (Draper and Ponsford, 2008) with concomitant psychosocial stress causing a higher prevalence of anxiety and depression (Miller et al., 2009). Psychological stress increases pro-inflammatory cytokines production, promotes rapid leukocyte transmigration (Cole, 2008), and activates neuroendocrine pathways releasing glucocorticoids, catecholamines, and cytokines compromising physiological, immunologic, and behavioral outcomes in both humans and rodents (Wohleb et al., 2012). Besides, the monoamine hypothesis proposes that the serotonin (5-HT), norepinephrine (NE), dopamine (DA), and neuropeptide Y (NPY) pathways play a crucial role in the pathophysiology of depression and anxiety (Eaton et al., 2007; Mickey et al., 2011; Hamon and Blier, 2013) and may aggravate depression and anxiety (Brigitta, 2002; Aan Het Rot et al., 2009) and sensorimotor deficits (Geyer et al., 2001; Wurm et al., 2007). Previous work has demonstrated an important role for psychological stress on cellular expression of adhesion molecules on circulating leukocytes (Goebel and Mills, 2000). However, the mechanisms of ICAM-1-mediated neuroinflammation and cell death, and its functional outcome following TBI, are still not clear.

In the present study, using an *in vitro* human brain microvascular endothelial cells (hBMVEC) stretch injury and an animal model of fluid percussion injury (FPI), we elucidated the mechanisms by which oxidative stress, matrix metalloproteinase (MMP), and vascular endothelial growth factor (VEGF) signaling activate ICAM-1 and result in subsequent neuroinflammation and neurodegeneration that leads to sensorimotor deficits and psychological stress. We propose that the activation of ICAM-1 following TBI upregulates NF-kB and other inflammatory cytokines and activates the caspase-3 enzyme that upregulates neurodegenerative markers. By studying the regulation of neurotransmitters expression, we also demonstrated the biochemical mechanisms of ICAM-1-mediated sensorimotor deficits and psychological stress after TBI. In the present study, we used ICAM-1 knock-out (KO; *ICAM-1*^−/−^) mouse *in vivo* and ICAM-1 siRNA and ICAM-1 inhibitor A205804 to validate the role of ICAM-1 in regulating neuroinflammation and neurodegeneration after TBI. We conclude that ICAM-1 has a specific role in impairing sensorimotor function and enhancing psychological stress by regulating neuroinflammation and neurodegeneration via the pathway of LFA-1 or Mac-1 proteins.

## Materials and Methods

### Reagents

The primary antibodies used were listed in Table 1. All secondary Alexa Fluor-conjugated antibodies, terminal deoxynucleotidyl transferase dUTP nick end labeling (TUNEL) kit, and DAPI were purchased from Invitrogen. siRNA transfection kit was purchased from Santa Cruz Biotechnology Inc. VEGF (recombinant human VEGF, catalog #293-VE) and ICAM-1 recombinant protein (IRP; recombinant human ICAM-1/CD54 protein, catalog #ADP4-050) were purchased from the R&D Systems. TIMP1 (MMPs inhibitor, catalog #580-RT) was from Calbiochem; Ki8751 (VEGFR-2 kinase inhibitor, catalog #676484) was from Sigma-Aldrich. Apocynin (NADPH oxidase inhibitor, catalog #11976) and A205804 (ICAM-1 inhibitor; catalog #21252) were purchased from Cayman Chemicals.

**Table 1:** Details of the antibodies used for this study

Antibody	Dilution	Catalog number	RRID	Vendor
Anti-ICAM-1	WB: 1:1000 IF: 1:250	ma5407	AB_223596	Invitrogen
Anti-Glut-1	IF: 1:250	ab80024	AB_2190927	Abcam
Anti-NeuN	IF: 1:250	ab104224	AB_10711040	Abcam
Anti- VEGF-A	WB: 1:1000	ma5-13182	AB_10981661	Invitrogen
Anti-4HNE	WB: 1:1000	ab46545	AB_722490	Abcam
Anti-MMP-9	WB: 1:1000	ab76003	AB_1310463	Abcam
Anti-LFA-1	WB: 1:1000 IF:1:250	ab186873	Not available	Abcam
Anti-MMP-2	WB: 1:1000	87809S	RRID:AB_2800107	Cell Signaling Technology
Anti-VEGFR-2 (p-Tyr1059)	WB: 1:1000	3817s	RRID:AB_2132351	Cell Signaling Technology
Anti-VEGFR-2 (p-1175)	WB: 1:1000	2478	RRID:AB_331377	Cell Signaling Technology
Anti-caspase-3	WB:1:1000	9662S	RRID:AB_331439	Cell Signaling Technology
Anti-NF-kB p65	WB: 1:1000	3033	RRID:AB_331284	Cell Signaling Technology
Anti-cleaved-caspase3	WB: 1:1000 AP IHC: 1:250	MAB835	AB_2243951	R&D Systems
Anti-NPY	WB: 1:1000	MAB8517	Not available	R&D Systems
Anti-Annexin V	WB: 1:1000	PA5-27872	AB_2545348	Thermofisher
Anti-β-actin	WB: 1:1000	MA575739	AB_2545348	Thermofisher
Anti-DA D1 receptor (DAD1R)	WB: 1:1000	NBP2-16213	AB_2819252	Novus Biologicalalals
Anti-NE	WB: 1:1000 IF: 1:200	AB120	AB_90481	EMD Millipore
Anti-5-HT1A receptor (5-HT1AR)	WB: 1:1000	GTX104703	AB_1241307	GeneTex
Anti-vWF	IF:1:250	ab11713	AB_298501	Abcam
Anti-NOX1	WB: 1:1000	GTX103888	AB_1951012	GeneTex

### Endothelial cell culture

hBMVECs were grown in DMEM/F12 media (Invitrogen) containing 10 mm HEPES, 13 mm sodium bicarbonate (pH 7), 10 mmol/l L-glutamine and supplemented with 10% heat-inactivated fetal bovine serum (FBS; Thermo Fisher Scientific), penicillin and streptomycin (100 μg/ml each, Life Technologies), 1% amphotericin (Caisson Labs), endothelial cell growth supplement (ECGS; 50 mg/ml; BD Bioscience), heparin (100 mg/ml; Sigma-Aldrich Co, Ltd; Abdul Muneer et al., 2012, 2018). hBMVECs were plated on rat tail collagen Type I (0.1 mg/ml) coated six-well Bioflex culture plates (Flexcell International Corp) at a density of 250,000 cells/well (Patel et al., 2017; Abdul-Muneer et al., 2018). The cells were fed by changing the medium every 2 d and grown until tight monolayers were formed in ∼6–8 d.

### *In vitro* stretch injury and treatment conditions

Confluent cells cultured in the Bioflex six-well culture plates were subjected to a stretch injury (biaxial) with a pressure of ∼3.0 psi using an *in vitro* cell injury device, Cell Injury Controller II (Custom design and fabrication Inc.) as we and others described previously (Geddes-Klein et al., 2006; Salvador et al., 2013; Patel et al., 2017; Abdul-Muneer et al., 2018; Bhowmick et al., 2019a). For experiments with pharmacological treatments and gene manipulations, the hBMVECs were treated with A205804 (ICAM-1 inhibitor, 100 ng/ml), IRP (recombinant human ICAM-1/CD54 protein, 2.5 μg/ml), apocynin (inhibitor of NADPH oxidase, 100 mm), VEGF-A (100 ng/ml), Ki8751 (VEGFR-2 kinase inhibitor, 10 μmol/l), Timp-1 (MMPs inhibitor, 100 ng/ml) 30 min before the stretch injury. For siRNA treatment groups, hBMVECs were transfected either with ICAM-1 siRNA (catalog #sc29354) or control siRNA (catalog #sc-37007) as per the manufacturer’s protocol (Santa Cruz Biotechnology). Before 30-min stretch injury (3 psi), the cells were treated with A205804 and IRP. For control siRNA and ICAM-1 siRNA transfection, the cells were incubated for 6 h in transfection media followed by replacing the transfection media with 1× normal growth media before the injury. Control hBMVECs were also cultured in the stretch injury culture plates but were devoid of stretch injury (Patel et al., 2017). At 24 h postinjury, cells were extracted and lysed in lysis buffer and proteins were extracted for western blotting. Subsequently, the supernatant was also collected for cytokine analysis.

### Animals and FPI

Male C57/BL6 wild-type (WT) and *ICAM-1*^−/−^ (ICAM-1 KO) mice (nine weeks old, 20–25 g; The Jackson Laboratory) were used for this study. Animals were maintained in sterile cages under pathogen-free conditions in accordance with institutional ethical guidelines for the care of laboratory animals, the National Institutes of Health, and the Seton Hall University Institutional Animal Care and Use Committee. We use a diurnal 12-h light cycle in our animal facility, and we conducted surgeries for FPI during the light cycle. To avoid the potential confounding effect of social heterogeneity in mixed-group housing of WT and KO littermates that might induce changes in each other’s behavior based on group inequality, and in a particular social hierarchy, on behavioral and physiological measures, we used independent non-littermate WT mice as controls.

**Figure 1.**
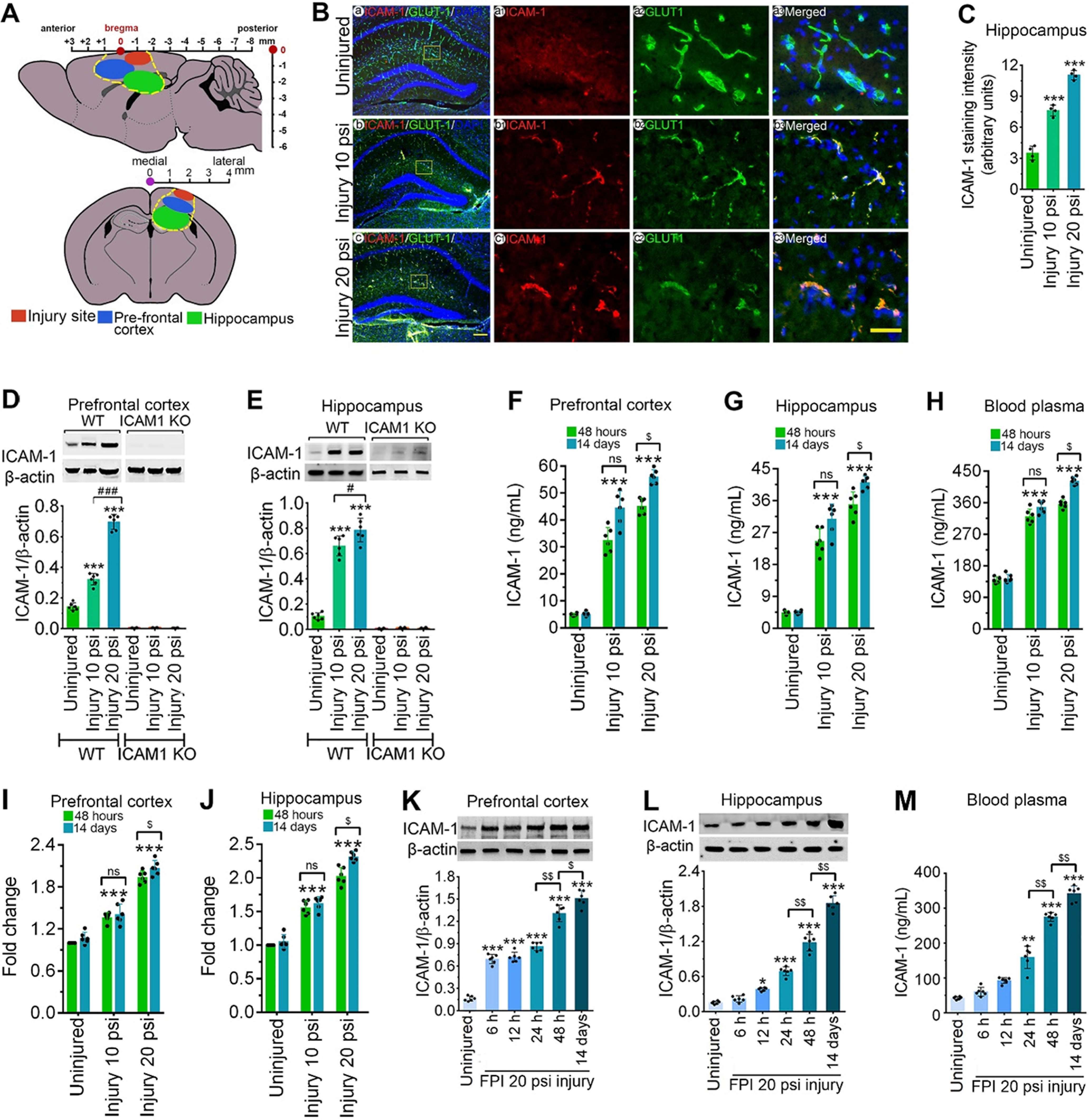
TBI activates ICAM-1 protein in both mild and moderate injury. ***A***, Representative illustration of injury site and tissue sampling area in the mice brain subjected to FPI. The injury site, PFC, and hippocampus are labeled with red, blue, and green colors, respectively. ***B***, Immunofluorescent staining of ICAM-1 (red) in the hippocampus area and merged with GLUT-1 (green) and DAPI (blue) after 10 and 20 psi FPI. Scale bar: 100 μm (shown in ***c*** is for ***a–c***, first column) and 40 μm (shows in ***c3*** is for ***a1–a3***, ***b1–b3***, and ***c1–c3***, columns 2–4). ***C***, Quantification of ICAM-1 staining in the hippocampus area of WT uninjured, 10 and 20 psi FPI mice using ImageJ software (*n* = 4/group). ***D***, ***E***, Western blot analysis of ICAM-1 and β-actin in the tissue lysates from PFC and hippocampus of WT and *ICAM-1*^−/−^ mice 48 h after 10 and 20 psi FPI. The bar graph with dot plots shows the quantification of ICAM-1 versus β-actin (*n* = 6/group). ***F–H***, ELISA quantification of ICAM-1 in PFC (***F***), hippocampus (***G***) tissue lysates and blood plasma (***H***) 48 h and 14 d following 10 and 20 psi FPI (*n* = 6/group). ***I***, ***J***, mRNA expression level of ICAM-1 using qPCR from PFC and hippocampus of WT and *ICAM-1*^−/−^ mice 48 h and 14 d after 10 and 20 psi FPI (*n* = 6/group). ***K***, ***L***, Western blot analysis of ICAM-1 and β-actin expression at different time points (6 h, 12 h, 24 h, 48 h, and 14 d) in the PFC (***K***) and hippocampus (***L***) of WT uninjured and 20 psi FPI. Bar graph represents the densitometric ratio of ICAM-1 bands versus β-actin bands (*n* = 6/group). ***M***, ELISA quantification of ICAM-1 at different time points (6 h, 12 h, 24 h, 48 h, and 14 d) in the blood plasma of WT uninjured and 20 psi FPI mice (*n* = 6/group). All values are expressed as mean ± SD. Statistically significant *p < 0.05, **p < 0.01, ***p < 0.001 versus WT uninjured group; #*p* < 0.05, ###*p* < 0.001 between 10 and 20 psi; $*p* < 0.05, $$*p* < 0.01 between time points; ns = non-significant.

Standard surgical methods for lateral FPI were performed in nine-week-old male mice (Kabadi et al., 2010; Abdul-Muneer et al., 2017b; Patel et al., 2017). We used male mice for this study and excluded female mice to avoid the potential effect of estrogen that might have anti-inflammatory properties by repressing the expression of ICAM-1 (Hou and Pei, 2015). Although sex-specific hormones are often associated with beneficial effects after TBI in animal models, translation to human studies has not been shown beneficial in any clinical trial (Stein et al., 2015). Briefly, 24 h before the injury, the animals were anesthetized with ketamine/xylazine mixture (80 mg/kg ketamine and 10 mg/kg xylene, i.p.) and surgically implanted with a Luer-Lok syringe hub to the right side of the skull in a stereotaxic device. This hub surrounds a craniotomy of the same size, positioned 1.0 mm posterior from and 2.0 mm lateral from the bregma (Fig. 1*A*). An additional cap that surrounds the syringe hub was applied. Two screws were implanted into the skull for additional support. The syringe hub was firmly fixed using the cranioplastic cement (AM Systems). Rectal temperatures were continuously monitored and maintained within normal ranges during surgical preparation by a feed-back temperature controller pad (model TC-1000; CWE Inc); 5% isoflurane was used to anesthetize the mice and anesthetize was confirmed when the foot-pinch reflex stopped. The animal was connected to a digitally controlled FPI system-FP302 (AmScien Instruments), and the injury was applied at 10 psi (mild) and 20 psi (moderate) with a pressure rise time of 8 ms. Animals exhibited apnea, loss of consciousness, and hyperextension of the tail and hind limbs after the injury. We observed a similar duration of transient apnea following moderate injury in both WT and ICAM-1 KO mice and no mortality rate was evident following transient apnea and all animals survived 48 h and 14 d post-TBI. The duration of apnea persists for ∼15–25 s followed by restoration of regular respiration and all animals gained consciousness within 35–40 s indicating that the groups had similar injury severity at the onset. Similarly, control mice received the same anesthesia and surgery as the injured group and connected to the dc-FPI without the injury. Six animals were used in each group and sample sizes for statistically significant results were prospectively derived by conducting power analyses using G*Power (University of Dusseldorf, Germany) based on our previous observations with outcome variations and effect sizes in the mouse model (Abdul-Muneer et al., 2013, 2017b). Our sample sizes were determined with the condition of an 80% chance of detecting a moderate effect size. Ketamine/xylazine mixture was used to anesthetize the animals after 6 h, 12 h, 24 h, 48 h, and 14 d postinjury and transcardially perfused with 1× PBS and 4% paraformaldehyde (PFA).

The *in vivo* study was conducted in the prefrontal cortex (PFC) and hippocampus because these two regions of the brain are considered part of the limbic system (Amunts et al., 2005; Morgane et al., 2005) and are vulnerable to TBI (Gray and McNaughton, 2000) and involve in sensorimotor and cognitive functions. Brain tissues from the PFC and hippocampus were collected and stored. Similarly, blood was drawn from the tail vein, separated blood plasma, and used for ELISA analysis. For immunostaining and TUNEL experiments, the brain tissues were embedded in an optimal cutting temperature (OCT) compound, stored frozen until analysis. Figure 1*A* shows the injury site and tissue sampling area in the mice brain cortex.

### Immunostaining and microscopy

#### Immunofluorescence

Immunofluorescence staining was performed in both cultured endothelial cells (on silicone membrane) and 10-μm-thick coronal brain sections as previously described (Abdul Muneer et al., 2012, 2013). Cells were washed with 1× PBS and fixed using 4% PFA for 20 min at 25°C. The coverslip culture cells and cryostat sections were then immersed in 1× PBS (pH 7.4) for 5 min, and then blocked in 3% normal goat serum containing 0.3% Triton X-100 in 1× PBS (pH 7.4) for 1 h at room temperature and later incubated with the primary antibodies overnight at 4°C. All antibodies were used at a concentration of 3.0 μg/ml. Cells were then washed in 1× PBS at room temperature for 15 min and incubated with secondary antibodies [Alexa Fluor 488 or 594 conjugated with anti-mouse or anti-rabbit immunoglobulin G (IgG); 1:500 dilution or 4 μg/ml] for 1 h and mounted with 5- to 10-μl immunomount containing DAPI (Invitrogen) on a slide. Semi-quantitative analysis of the protein of interest was done as previously described (Abdul Muneer et al., 2012, 2018), and images were captured using the Eclipse TE200 fluorescent microscope (Nikon) and NIS Elements software (Nikon) in three channels, DAPI (cell nuclei, blue color emission), Alexa Fluor 488 (green color emission), and Alexa Fluor 594 (red color emission). For imaging, the area of image capture was randomized and was performed by a researcher that was blinded to the experimental conditions. The analysis was performed on at least four samples for immunofluorescent staining and captured six images from a single sample (slide). For consistency during image capture, the same parameters of camera and software including the brightness of the excitation light, the detector sensitivity (gain), or the camera exposure time across the samples were used. The intensity of immunostaining was analyzed by ImageJ (NIH) software (Bankhead, 2014). The uneven illumination in fluorescence images was corrected by keeping a uniform dark background for all the images.

#### Alkaline phosphatase (AP) immunohistochemistry

Ten-micrometer thickness PFA-fixed coronal brain tissue sections were washed in 1× PBS and incubated with 0.3% H_2_O_2_ in 1× PBS and 0.3% Triton X-100 for 20 min at room temperature. Then the tissue slides were rinsed three times in 1× PBS followed by blocking the tissues in 2% normal goat serum in phosphate buffer containing 0.3% Triton X-100 for 20 min at room temperature. After three times wash with 1× PBS, the tissue sections were incubated with primary antibody rabbit anti-cleaved-caspase-3 (cl-caspase-3) with 0.3% Triton X-100 overnight at 4°C. The primary antibody was rinsed off three times with 1× PBS, and the tissues were incubated with 0.5 ml of AP-conjugated secondary antibody (1:500 dilution in blocking reagent) for 1 h followed by two washes with 1× PBS and once with assay buffer of AP substrate (100 mm Tris-Cl, pH 9.5), 5 min each. Then applied 500 μl of AP substrate reagent to each tissue section followed by incubation in dark for 20–30 min at room temperature. After washing, the tissue slides were mounted with histomount and glass coverslips for microscopy analyses.

### Western blotting

Protein lysates from *in vitro* and *in vivo* experiments were prepared as we described previously (Abdul-Muneer et al., 2018; Bhowmick et al., 2019b). Briefly, the cells (three wells of the same condition) and tissues (50 mg cortical tissue below the injury from the neo-cortex area) were lysed with Cell Lytic-M buffer (Thermo Scientific) containing a mixture of mini protease inhibitor cocktail tablets (Sigma-Aldrich) and protein were extracted. Protein concentration was measured by the bicinchoninic acid (BCA) assay kit (Thermo Scientific). Immunoblots were performed by resolving the protein (10 μg) in 4–15% gradient SDS-PAGE gel (Bio-Rad), and the blots were transferred onto a nitrocellulose membrane and blocked with (5%) dry milk for 1 h at room temperature. The membranes were incubated overnight at 4°C with primary antibodies (1 μg/ml) against ICAM-1, VEGF-A, VEGFR-2, 4HNE, MMP-2, MMP-9, LFA-1, pVEGFR2^Tyr1059^, pVEGFR2^Try1175^, caspase-3, NFkB p65, cl-caspase-3, Annexin V, DAD1R/DRD1R, NE, 5-HT, 5-HT1AR, NPY, and β-actin antibodies. After washing three times at 10-min intervals, the membranes were incubated for 1 h at room temperature with horseradish peroxidase-conjugated secondary antibodies (1:5000; Fisher Scientific). The membrane was rinsed three times with TBS-Tween for 10 min at room temperature. Protein bands were detected using chemiluminescence western blot detection reagents (Advansta) and scanned with an imager, Syngene gel documentation system. The optical density was quantified as arbitrary densitometry intensity units using the ImageJ software package (NIH). The protein of interest was normalized and quantified using β-actin as a loading control.

### Quantitative real-time PCR (qRT-PCR)

The mRNA expression levels of ICAM-1 was determined by qRT-PCR. Following 48 h and 14 d after mild (10 psi) and moderate (20 psi) FPI, WT, and *ICAM-1*^−/−^ mice were euthanized and the brains were removed and stored at −80°C. Total RNA from the injured PFC and hippocampus was extracted using Qiagen RNeasy Mini kit (QIAGEN, catalog #74104) following the manufacturer’s protocol followed by DNase I treatment for 20 min at 37°C to remove genomic DNA contamination. The quantity, purity, and integrity of the RNA were evaluated using Qubit 2.0 (Thermo Fisher Scientific) and Agilent Bioanalyzer (Agilent Technologies). Isolated total RNA from the sample was then converted to cDNA using the iScript cDNA synthesis kit (Bio-Rad). The qPCR was performed using these primers: mouse ICAM-1 (forward, 5′-CAATTTCTCATGCCGCACAG-3′; reverse, 5′-AGCTGGAAGATCGAAAGTCCG-3′), mouse GAPDH (forward, 5′-GGTCGGTGTGAACGGATTT-3′; reverse, 5′-GTGGATGCAGGGATGATGTT-3′). PCR amplification was performed with 25 ng of starting cDNA material mixed with gene-specific forward and reverse primers and iTaq Universal (Bio-Rad Laboratories) in a 20 μl reaction on a StepOne Real-Time System (Applied Biosystems) with the PCR conditions: 95°C for 10 min, 95°C for 30 s, 55°C for 1 min, 72°C for 1 min for 40 cycles and then 95°C for 1 min, 55°C for 30 s, and 95°C for 30 s. GAPDH mRNA was quantified as an endogenous control. Fold-change differences across groups were determined using the ΔΔCt method.

### Co-immunoprecipitation (IP)

Co-IP was performed to analyze the ICAM-1, LFA-1, and Mac-1 protein-protein interactions using Pierce Classic Magnetic IP/Co-IP kit (catalog #88804). Briefly, brain tissue (50 mg) from WT and *ICAM-1*^−/−^ mice with and without FPI were harvested and lysed with 500 μl of IP Lysis/Wash buffer and following lysis and centrifugation, the supernatant was transferred to a new tube for protein concentration determination. For this experiment, Pierce Protein A/G Magnetic Beads were used for IP according to the manufacturer’s protocol. Briefly, 500 μg of total protein extract per sample was subjected to IP assay using 10 μg of anti-ICAM-1 antibody and incubated overnight at 4°C. After the formation of the immune complex, A/G Magnetic Beads were added and incubated for 1 h. After washing the IP complexes, the samples were suspended in 200 μl of 2xSDS sample buffer. Twenty microliters of aliquots were then subjected to Western blotting using ICAM-1, LFA-1, and Mac-1 antibodies.

### *In vitro* cell transmigration across the BBB

To quantitatively analyze the changes in cell adhesion and monocytes traffic across the altered BBB, hMBECs (2.5 × 10^4^ cells/insert) were cultured on Type I collagen-coated FluoroBlok tinted tissue culture inserts (with 3-μm pores, BD Biosciences). After the formation of the tight monolayer, cell cultures were treated with A205804, IRP, ICAM-1 siRNA, or control siRNA as per the manufacturer’s protocol (see above, *In vitro* stretch injury and treatment conditions). Then cell tracker Fluo3 (a green fluorescence cell tracker; 5 μm, Invitrogen)-labeled primary human macrophages were introduced into the endothelial monolayers (1 × 10^6^ monocytes/insert). Cells were then allowed to infiltrate into the monolayers for 2 h at 37°C in tissue culture incubator in the absence of test compounds. The relative fluorescence intensity of the migrated cells at the lower chamber and those cells that were stuck between the porous membranes and the lower chambers were detected by a fluorescence-based assay using the Tecan Genios fluorescence plate reader. The actual numbers of migrated cells were calculated from the internal standard curve of the labeled cells, and the data were presented as the fold difference of the untreated control.

### *In vivo* cell adhesion and migration assay

As we previously reported (Abdul-Muneer et al., 2017a), femoral bones from euthanized mice were dissected out under sterile conditions and washed in 1× PBS. Bone marrow was flushed out repeatedly with 1× HBSS through the cut ends of the bones using a 1-ml syringe. The bone marrow suspension was filtered through a 40-μm cell strainer. The filtrate was collected and centrifuged at 1, 800 rpm for 5 min at 4°C. The pellet was suspended in 1 ml of DMEM/F-12 media containing 10% FBS, penicillin and streptomycin (100 μg/ml each, Invitrogen) and 0.001% macrophage-colony stimulating factor (MCSF; 500 μl in 500-ml media). Then the cells were dissociated by trituration (10–15 times) and counted with Trypan Blue using Hemocytometer. Bone marrow-derived cells were differentiated to macrophages in culture containing the MCSF in the culture medium (plating 2 × 10^6^ cells/T-75 flask). Cells were fed every third day. After 6 d, the differentiated macrophages were detached by a cell scraper, suspended in 1 ml HBSS and centrifuged at 1800 rpm for 5 min at 4°C. Cell pellets were then dissociated by repeated trituration, counted and were labeled with Fluo3 (Invitrogen, Invitrogen) in 1 ml of HBSS at 37°C for 15 min. Excess unbound Fluo3 was washed out by centrifugation and cell pellets resuspended in HBSS (2 × 10^6^ cells) was infused through the common carotid artery using 30-G needle. The animal was euthanized 1 h after the cell infusion and brain microvessels were surgically removed for observation of calcein AM-labeled macrophages under the fluorescent microscope.

### ELISA

Using commercial ELISA kits, the level of IL-1β, and TNF-α (Life Technologies-Thermo Fisher Scientific; Abdul Muneer et al., 2012, 2018) and ICAM-1 (Abcam) were analyzed in cell culture media, cell lysates, tissue lysates from PFC and hippocampus, and blood plasma as per the manufacturer’s instructions.

### Analysis of cerebral blood flow (CBF) and lesion volume

CBF was monitored over the cortices ipsilateral to the injury before the injury to establish a baseline and 48 h after experimental FPI injury using a Perimed laser doppler flowmeter (LDF) device (Periflux 5000, Perimed AB). Briefly, the optical brain probe was placed (−1 mm anterior-posterior and +2 mm lateral to the bregma) and was installed on the stereotaxic frame to reduce the influence of movements that otherwise would affect the recorded signals. The probe was kept at the same skull site for at least 10 s up to several minutes to receive stable signals. Two signals output is extracted from the LDFM device: (1) the total backscattered light (TLI), which represents the diffuse reflection of light and is related to the tissue type; and (2) the perfusion (microvascular blood flow), which represents relative changes in the tissue’s microcirculation. The total range of the TLI and perfusion signals are presented as 0–10.0 arbitrary units (a.u) and 0–999 a.u., respectively. For all measurements, the data acquisition sampling frequency (fs) was set to 100 Hz. The probe was tested in Motility Standard solution (Perimed AB) before each measurement to verify comparable perfusion and TLI levels between measurements. Laser Doppler blood flow was measured as perfusion units and normalized to the preinjury baseline.

Lesion volume was measured as previously reported (Villapol et al., 2015).

### TUNEL and trypan blue histologic staining

Using the TUNEL (Invitrogen-Thermo Fisher Scientific) assay kit, cell apoptosis was determined in fixed cultured cells and tissue sections as per the manufacturer’s instructions (Patel et al., 2017; Abdul-Muneer et al., 2018). Briefly, coronal brain tissue sections (10-μm thickness) fixed with 4% PFA were incubated with DNA labeling solution containing reaction buffer, terminal deoxynucleotidyl transferase (TdT) enzyme, and BrdUTP for 1 h at 37°C. The tissue slides were rinsed three times (5 min each) in rinse buffer with gentle shaking and then incubated with Alexa Flour 488 dye-conjugated anti-BrdU antibody for 30 min in the dark. The slides were then incubated with propidium iodide/RNase A staining buffer for 30 min at room temperature in the dark. The slides were then rinsed with wash buffer for 5 min and after drying mounted with DAPI for imaging. For analyzing the dead cells, trypan blue staining was performed to determine the percentage of dead cells 24 h after 2.0 psi stretch injury in the cell lysates of hBMVEC treated with control siRNA, ICAM-1 siRNA, A205804, and IRP.

### Behavioral studies

To measure the immediate effect of TBI on behavioral outcomes in male C57/BL6 WT and *ICAM-1*^−/−^ (ICAM-1 KO) mice, behavioral tests were performed on day 0 (baseline), 48 h, and 14 d postinjury. All behavioral tests were performed within the 12/12 h light/dark cycle, and experimenters were blinded to the treatment groups as described previously (Bhowmick et al., 2018). For monitoring motor functions, rotarod and grid walk test were performed. The sucrose preference test was used to analyze depression and the light-dark box test was used to analyze anxiety.

#### Rotarod test

Briefly, for the rotarod test, the latency to fall on the rotarod was monitored using the rotarod system by trained personnel blinded to animal groups as described (Liu et al., 2018). Animals were pretrained on an automated four-lane rotarod unit and the task was performed on day 0 (baseline), 48 h, and 14 d after FPI. The animal was placed on the rod and tested for its latency to fall time. The starting speed was set to 0, and the speed was increased by 2 rpm every 5 s up to 40 rpm. Total time in seconds that the animal could stay on the rod was recorded. Each animal was given four trials, and the mean latency to fall from three trials was calculated for each animal.

#### Grid-walk test

For the grid walk test, mice were placed on an elevated metallic grid (45 × 100 cm) having 1.0-cm separation between two grids and allowed to walk for 1 min while being videotaped from below, as previously described (Bhowmick et al., 2018). Videos were later analyzed for total walking time and the number of forelimb foot errors for each foot. The grid walk errors were counted (four separate trials per test) and averaged from the trials. Anxiety-like behavior was assessed using the sucrose preference test and light-dark box test (Bourin and Hascoët, 2003; Boccella et al., 2020).

#### Sucrose preference test

For the sucrose preference test, all mice were habituated to the presence of two drinking bottles (one containing 1% sucrose and the other water) for 3 d in their home cage. Following this acclimation, mice have the free choice of either drinking the 1% sucrose solution or plain water, and the positions of two bottles are switched daily to reduce any confound produced by a side bias. The quantities of pure water and sucrose consumed were recorded at baseline, 48 h, and 14 d. Sucrose preference was measured as: sucrose preference percentage (%) = sucrose solution consumption (g)/[sucrose solution consumption (g) + water consumption (g)] × 100%. The quantities of pure water and sucrose consumed were recorded at baseline, and again at 48 h and 14 d post-TBI.

#### Light-dark box test

For the light-dark test, the apparatus (60 × 30 × 30 cm; length × width × height) is divided into two equally sized compartments: the dark chamber was covered, whereas the light compartment was opened brightly illuminated (∼150 lux). The two boxes were separated by a guillotine door. Mice were placed in the light compartment and allowed to explore between the two chambers for 10 min. The time spent in each compartment and the number of transitions were recorded.

### Data analysis

Statistical analyses of the data were performed using GraphPad Prism V7. All the results are expressed as the mean ± SD. For data with a normal distribution, as tested with Kolmogorov– Smirnoff tests, one-way (treatment) or two-way ANOVA (treatment × strain) with and without repeated measures (time) were performed wherever applicable to obtain significant main effect followed by Bonferroni *post hoc* tests to determine the significant differences between controls and experimental conditions within and between strains, and a probability value of *p* < 0.05 was considered significant.

## Results

### ICAM-1 expression is upregulated after FPI *in vivo* and *in vitro* stretch injury in a time-dependent manner

We first elucidated the spatial and temporal distribution of ICAM-1 in WT and *ICAM-1*^−/−^ mice subjected to 10 psi (mild) and 20 psi (moderate) FPI. Figure 1*A* shows the area of interest and FPI-induced injury and penumbra sites (PFC: −0 to +2.5 to bregma, hippocampus: −0.5 to −3 to bregma) used for evaluation. Immunofluorescence staining in the hippocampus revealed a marked increase in ICAM-1 expression in 10 and 20 psi injury than the WT uninjured group and are in colocalization with the endothelial cell marker, GLUT-1 (*F*_(2,9)_ = 186.1, *p* = 0.0001; Fig. 1*B*,*C*). Similarly, the ICAM-1 was significantly expressed and colocalized with the endothelial cell marker, von Willibrand factor (vWF) in the PFC subjected to 10 and 20 psi FPI (data not shown). Further validation of ICAM-1 expression in the PFC and hippocampus using western blotting revealed a significant increase in ICAM-1 expression in both 10 and 20 psi FPI WT mice consistent with the immunofluorescence analysis, however, in ICAM-1^−/−^ mice, a negligible level of ICAM-1 protein was observed in both 10 and 20 psi FPI without any significant difference in the expression pattern of ICAM-1 when compared with uninjured *ICAM-1*^−/−^ group (PFC: *F*_(2,30)_ = 331.6, *p* = 0.0001; hippocampus: *F*_(2,30)_ = 149.4, *p* = 0.0001; Fig. 1*D*,*E*). The temporal expression of ICAM-1 in tissue lysates from the PFC (*F*_(2,14)_ = 15.46, *p* = 0.0002), hippocampus (*F*_(2,15)_ = 7.078, *p* = 0.0068), and blood plasma (*F*_(2,15)_ = 25.19, *p* < 0.0001) shows a significant increase in the level of ICAM-1 at 14 d compared with 48 h postinjury in only 20 psi (moderate) injury groups but not in 10 psi (mild) injury group (Fig. 1*F–H*) indicating a differential expression of ICAM-1 correlates to the level of injury (mild vs moderate) and time. In context to the transcriptional profile of ICAM-1, a significant upregulation of ICAM-1 mRNA in the PFC (*F*_(1,15)_ = 10.73, *p* = 0.0051) and hippocampus (*F*_(2,15)_ = 10.1, *p* = 0.0017) was observed in injured samples (Fig. 1*I*,*J*). Although the 20 psi FPI group showed significant upregulation of ICAM-1 mRNA between 48 h and 14 d FPI, there was no significant change in ICAM-1 mRNA expression in the 10 psi FPI group when compared between 48 h and 14 d post-FPI (Fig. 1*I*,*J*). When the altered expression of constitutively expressed ICAM-1 was examined at various times following FPI, tissue lysates from both PFC (*F*_(5,25)_ = 2.759, *p* = 0.0406) and hippocampus (*F*_(5,25)_ = 2.883, *p* = 0.0307) displayed a significant increase in the expression of ICAM-1 protein level at 6 h, 12 h, 24 h, 48 h, and 14 d as compared with uninjured group (Fig. 1*K*,*L*). Although in both PFC and hippocampus we did not notice any significant increase in ICAM-1 protein expression between 6 h and 12 h, 24 h, 48 h, and 14 d post-TBI causes a significant induction in ICAM-1 protein expression in 20 psi injured samples. Interestingly, a significant change in ICAM-1 protein expression was noticed between 24 and 48 h and 48 h and 14 d post-TBI injury (Fig. 1*K*,*L*). When blood plasma was analyzed, the trend in the expression level of ICAM-1 was similar to the PFC and hippocampus (*F*_(5,25)_ = 304.5, *p* < 0.0001; Fig. 1*M*).

**Figure 2.**
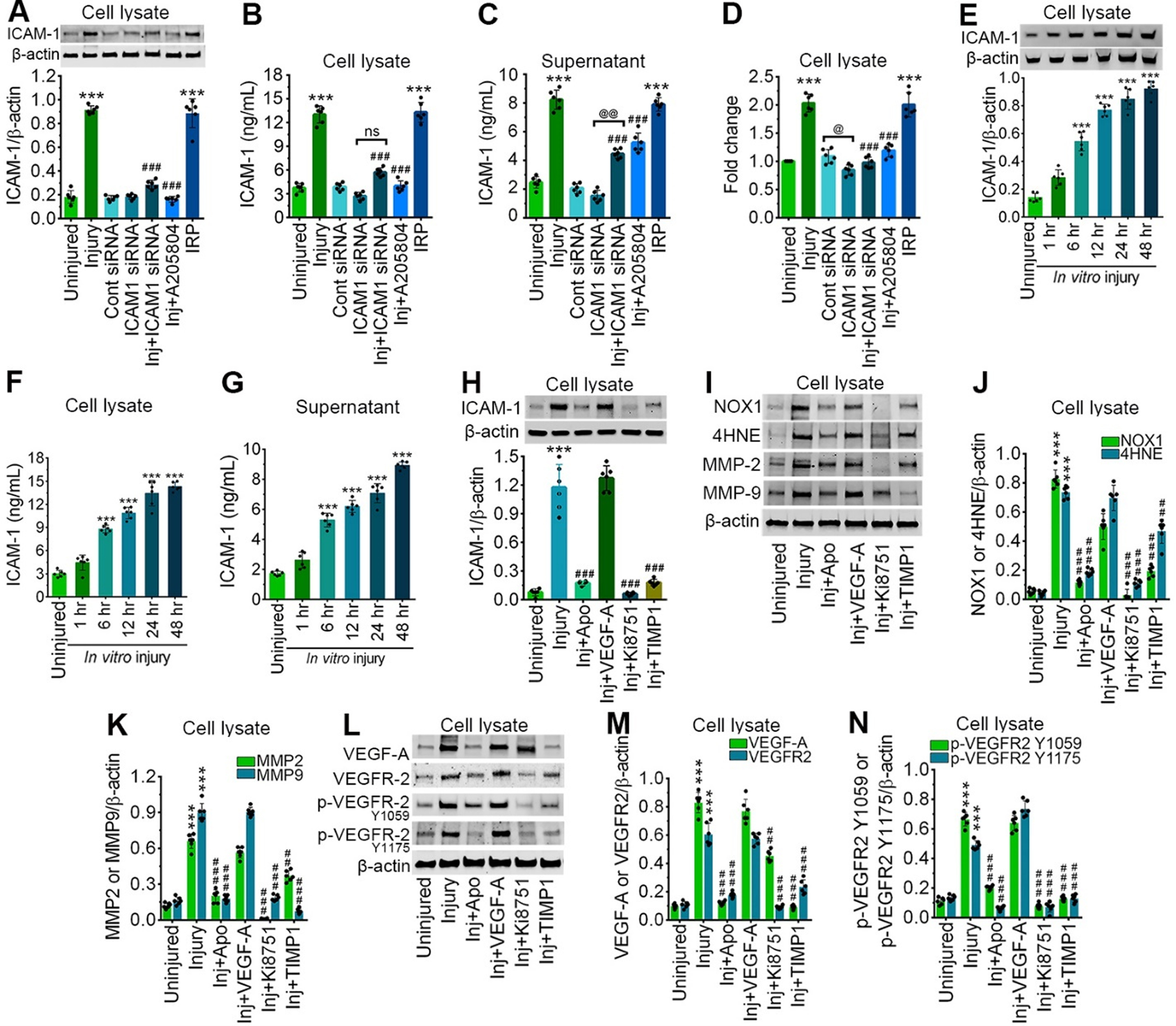
Stretch injury causes the activation of ICAM-1 in a time-dependent manner and is regulated by oxidative stress, MMPs, and VEGF signaling. ***A***, Western blot analysis of ICAM-1 and β-actin 24 h after 3.0 psi stretch injury in the cell lysates of hBMVEC treated with control siRNA, ICAM-1 siRNA, A205804, and IRP. Bar graph represents the quantification of ICAM-1 versus β-actin (*n* = 6/group). ***B***, ***C***, ELISA quantification of ICAM-1 in cell lysates (***B***), and cell culture supernatant (***C***) of hBMVEC following 3.0 psi stretch injury treated with control siRNA, ICAM-1 siRNA, A205804, and IRP (*n* = 6/group). ***D***, mRNA expression level of ICAM-1 using qPCR from hBMVEC treated with control siRNA, ICAM-1 siRNA, A205804, and IRP (*n* = 6/group) 24 h after 3.0 psi stretch injury. ***E*,** Western blot analysis of ICAM-1 and β-actin expression at different time points (1, 6, 12, 24, 48 h) in hBMVEC lysates after 24 h 3.0 psi stretch injury. Bar graph represents the quantification of ICAM-1 versus β-actin (*n* = 6/group). ***F***, ***G***, ELISA quantification of ICAM-1 in cell lysate (***F***), and cell culture supernatant (***G***) of hBMVEC at different time points (1, 6, 12, 24, 48 h) following 3.0 psi stretch injury. ***H***, Western blot analysis of ICAM-1 and β-actin 24 h after 3.0 psi stretch injury in the cell lysates of hBMVEC treated with apocynin (NADPH oxidase inhibitor), VEGF-A (recombinant human VEGF), Ki8751 (inhibitor of VEGFR phosphorylation), and TIMP1 (MMPs inhibitor). Bar graph represents the quantification of ICAM-1 versus β-actin (*n* = 6/group). ***I–K***, Western blot analysis of NOX1, 4HNE, MMP-2, MMP-9, and β-actin 24 h after 3.0 psi stretch injury in the cell lysates of hBMVEC treated with apocynin, VEGF-A, Ki8751, and TIMP1. Bar graph represents the quantification of ICAM-1 versus β-actin (*n* = 6/group). ***L–N***, Western blot analysis of VEGF-A, VEGFR-2, p-VEGFR-2^Y1059^, p-VEGFR-2^Y1175^, and β-actin 24 h after 3.0 psi stretch injury in the cell lysates of hBMVEC treated with apocynin, VEGF-A, Ki8751, and TIMP1 (*n* = 6/group). All values are expressed as mean ± SD. Statistically significant, ***p < 0.001 versus uninjured group; ##*p* < 0.01, ###*p* < 0.001 versus injury group.

Next, using an *in vitro* approach, we validated the regulatory mechanisms of ICAM-1 expression. Our western blotting result shows that hBMVEC cells subjected to 3 psi stretch injury incurred a ∼5.0-fold increase in the expression of ICAM-1 protein 24 h after injury, however, pretreatment with ICAM-1 siRNA or ICAM-1 inhibitor (A205804) in injured cells markedly reduced the ICAM-1 expression (*F*_(6,35)_ = 234.7, *p* < 0.0001; Fig. 2*A*). ELISA analysis in cell lysates (*F*_(6,35)_ = 209.1, *p* < 0.0001) and in the cell-supernatant (*F*_(6,35)_ = 189, *p* < 0.0001) further validated a similar reduction in the expression of ICAM-1 with ICAM-1 siRNA or ICAM-1 inhibitor pretreatment (Fig. 2*B*,*C*). Evaluation of ICAM-1 expression with the treatment of positive control, IRP further confirms an increase in the expression of ICAM-1 similar to what was observed in the injury group (Fig. 2*A–C*). Similarly, the level of ICAM-1 mRNA expression analyzed by qRT-PCR shows a trend similar to the ICAM-1 protein expression (*F*_(6,35)_ = 88.97, *p* < 0.0001; Fig. 2*D*). Next, we examined the temporal resolution of ICAM-1 expression level at 1, 6, 12, 24, and 48 h following 3 psi stretch injury in both cell lysates and supernatant. Western blotting data showed a significant increase in the expression pattern of ICAM-1 protein from 6 h until 48 h after stretch injury. The temporal resolution was conducted until 48 h since no significant change was observed between 24 and 48 h (*F*_(5,30)_ = 195.2, *p* < 0.0001; Fig. 2*E*). Likewise, ELISA data showed a similar trend in the increase in ICAM-1 levels during the time course of study in both cell lysates and supernatant from 6 h with a maximum increase in ICAM-1 expression at 48 h (cell lysate: *F*_(5,25)_ = 2.85, *p* = 0.0360; supernatant: *F*_(5,25)_ = 5.157, *p* = 0.0022; Fig. 2*F*,*G*).

### ICAM-1 expression is in part modulated by oxidative stress, MMPs, and VEGF signaling

Next, to decipher the upstream processes that possibly trigger the induction of ICAM-1 (Ksiazek et al., 2010; Dong et al., 2011), we investigated the role of oxidative stress (using apocynin, NADPH inhibitor), VEGF (using Ki8751, an inhibitor of VEGFR phosphorylation), and MMPs (using TIMP1, an MMP inhibitor) signaling in the regulation of ICAM-1 expression and subsequent activation of oxidative stress markers, MMPs, VEGF-A, and VEGFR-2 and its phosphorylated forms. Western blot analysis reveals that apocynin pretreatment causes a significant reduction in ICAM-1 expression in 3 psi stretch injured cells. Although exogenous VEGF-A induction with 3 psi injury shows no marked increase in ICAM-1 protein expression, inhibiting VEGF signaling with Ki8751 resulted in a significant decrease in ICAM-1 expression when compared with 3 psi injury alone. Similarly, MMP inhibitor, TIMP1 pretreatment showed a significant effect in alleviating the expression level of ICAM-1 (*F*_(5,30)_ = 148.9, *p* < 0.0001; Fig. 2*H*). Further evaluation of oxidative stress markers revealed a significant decrease in NOX-1 (*F*_(5,30)_ = 230, *p* < 0.0001) and 4HNE (*F*_(5,30)_ = 184.3, *p* < 0.0001) expression in the stretch injured group on pretreatment with apocynin, Ki8751, or TIMP1 inhibitor (Fig. 2*I–K*). A similar reduction in MMP2 (*F*_(5,30)_ = 214.2, *p* < 0.0001) and MMP9 (*F*_(5,30)_ = 534.6, *p* < 0.0001) protein expression was evident in stretch injured cells on pretreatment with apocynin, Ki8751, or TIMP1 inhibitor (Fig. 2*I–K*). Next, to analyze the role of VEGF signaling, we measured the expression levels of VEGF and its receptors. Although stretch injury, causes a marked increase in the expression levels of VEGF-A, VEGFR-2, and its phosphorylated forms (Y1059 and Y1175), no further increase was observed on exogenous VEGF-A treatment along with 3 psi injury. Interestingly, induction of apocynin, Ki8751 or TIMP1 inhibitor before injury resulted in a substantial alleviation in the expression levels of VEGF-A (*F*_(5,30)_ = 295.6, *p* < 0.0001), VEGFR-2 (*F*_(5,30)_ = 172.9, *p* < 0.0001), and its phosphorylated forms: Y1059 (*F*_(5,30)_ = 438.9, *p* < 0.0001) and Y1175 (*F*_(5,30)_ = 450.6, *p* < 0.0001; Fig. 2*L–N*).

### TBI augments the expression and cooperative interaction of ICAM-1 with its receptors LFA-1 and Mac-1 and promotes transmigration of leukocytes to the brain

**Figure 3.**
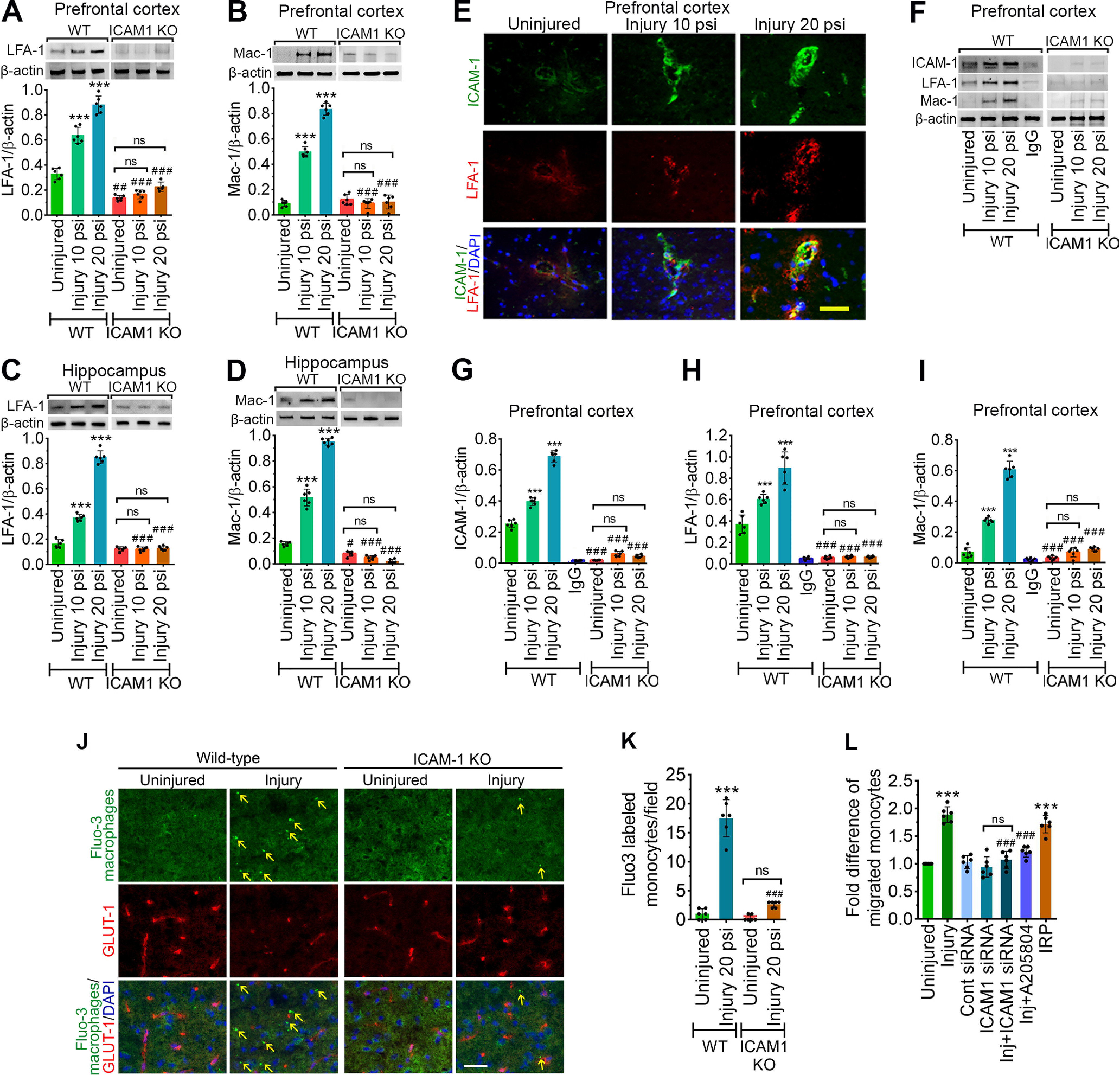
TBI enhances the activation of LFA-1 or Mac-1 with ICAM-1 and promotes transmigration of leukocytes. ***A***, ***B***, Western blotting of LFA-1 (***A***), Mac-1 (***B***), and β-actin following 10 and 20 psi FPI in the prefrontal cortical tissue lysates of WT and *ICAM-1*^−/−^ mice. Bar graph represents the densitometric ratio of LFA-1 or Mac-1 bands versus β-actin bands (*n* = 6/group). ***C***, ***D***, Western blotting of LFA-1 (***C***), Mac-1 (***D***), and β-actin in the hippocampal tissue lysates of WT and *ICAM-1*^−/−^ mice. Bar graph represents the densitometric ratio of LFA-1 or Mac-1 bands versus β-actin bands (*n* = 6/group). ***E***, Immunofluorescent staining of ICAM-1 (green) merged with LFA-1 (red) and DAPI (blue) in WT mice 48 h after 10 and 20 psi FPI (*n* = 4). ***F***, Western blotting of ICAM-1, LFA-1, Mac-1, and β-actin 48 h after 10 and 20 psi FPI in the tissue lysates immunoprecipitated with ICAM-1 mAb. Anti-IgG antibody was used as a negative control (fourth lane in WT blots). ***G–I***, Bar graphs show the densitometric ratio of ICAM-1 (***G***), LFA-1 (***H***), and Mac-1 (***I***) bands versus β-actin bands (*n* = 6/group). ***J–L***, *In vivo* and *in vitro* analysis of transmigration of leukocytes across the BBB. Fluo3-labeled macrophage adhesion/migration in brain capillary after infusion of cells into the common carotid artery (***J***), and the quantification of Fluo3-labeled cells in the brain part (***K***). ***L***, Migration of monocytes across the *in vitro* model of BBB after treatment of various test compounds as shown in figure (*n* = 6/group). All values are expressed as mean ± SD two-way ANOVA followed by Bonferroni *post hoc* tests. Statistically significant ***p < 0.001 versus WT uninjured group; ##*p* < 0.01, ###*p* < 0.001 uninjured versus uninjured, 10 versus 10 psi, and 20 versus 20 psi between WT and KO groups; ns = non-significant.

Since ICAM-1 is crucial in promoting adhesion and transmigration of circulating leukocytes and macrophages (Ding et al., 1999; Abdul Muneer et al., 2011; Desai et al., 2016), we next analyzed the effect of TBI in the expression of LFA-1 and Mac-1. In the PFC and hippocampus, compared with uninjured group, both 10 and 20 psi FPI causes a significant increase in LFA-1 (PFC: *F*_(2,29)_ = 65.63, *p* < 0.0001; hippocampus: *F*_(2,30)_ = 419.9, *p* < 0.0001) and Mac-1 (PFC: *F*_(2,30)_ = 246.1, *p* < 0.0001; hippocampus: *F*_(2,30)_ = 529.3, *p* < 0.0001) expression in WT groups. In contrast, when the PFC and hippocampus were analyzed, no significant change in the expression levels of LFA-1 (PFC: *F*_(2,3)_ = 0.4514, *p* = 0.6740; hippocampus: *F*_(2,3)_ = 0.653, *p* = 0.5815) and Mac-1 (PFC: *F*_(2,3)_ = 0.5589, *p* = 0.6218; hippocampus: *F*_(2,3)_ = 0.3736, *p* = 0.7163) was evident between the uninjured and injured *ICAM-1*^−/−^ mice (Fig. 3*A–D*). The validation of the interaction of ICAM-1 with LFA-1 and Mac-1 using immunofluorescence staining further confirms the colocalization of LFA-1 with ICAM-1 in brain microvessels and the intensity of colocalization increased in both 10 and 20 psi when compared with the uninjured group (Fig. 3*E*). Further co-IP of ICAM-1 with LFA-1 or Mac-1 provides strong evidence that injury in WT group results in a concomitant increase in LFA-1 (*F*_(2,30)_ = 37.12, *p* < 0.0001) and Mac-1 (*F*_(2,30)_ = 188.4, *p* < 0.0001) expression level with increased ICAM-1 expression (*F*_(2,30)_ = 271.5, *p* < 0.0001). ICAM-1 deletion resulted in a complete downregulation of both LFA-1 and Mac-1 protein expression despite the induction of injury further validating the strong interaction of ICAM-1 with LFA-1 or Mac-1 (Fig. 3*F–I*).

To validate the regulatory role of ICAM-1 in the transmigration of immune cells to the brain, we next infused Fluo3-labeled macrophages via the common carotid artery in WT, and *ICAM-1*^−/−^ animals 48 h following injury. Frontal cortical tissues were processed 2 h after macrophages infusion and analyzed for transmigration of macrophages to the brain. To show the brain microvessel, the tissue sections were stained with GLUT1, the endothelial cell-specific marker. Using fluorescence microscopy, we observed a significant number of Fluo3-positive cells in the cortical tissue part of the brain of the WT injured mice (*p* < 0.0001) compared with uninjured controls. However, injured *ICAM-1*^−/−^ mice resulted in preventing the leakage of Fluo3-positive macrophages to the abluminal part of the brain microvessel (*p* < 0.0001; Fig. 3*J*,*K*).

Since we observed the role of ICAM-1 in regulating transmigration of monocytes to the brain *in vivo*, we next sought to determine the mechanisms of regulation of monocyte transmigration in injured hBMVECs. Infiltration of immune cells into the brain was assayed by adhesion and migration of Fluo3 (a green fluorescence cell tracker)-labeled macrophage using an *in vitro* BBB model. A 2-fold increase in macrophage transmigration was observed with stretch injury as compared with uninjured control cells (*F*_(6,35)_ = 178.3, *p* < 0.0001). However, pretreatment with ICAM-1 siRNA or ICAM-1 inhibitor (A205804) in injured cells markedly reduced the macrophage transmigration (Fig. 3*L*). As expected, when the cells were induced with IRP, we observed a sharp increase in macrophage transmigration (*F*_(6,35)_ = 142.7, *p* < 0.0001). This indicates that ICAM-1 has significant role in transmigration of leukocytes following brain injury.

### TBI reduces CBF in the pericontusional area after FPI injury

**Figure 4.**
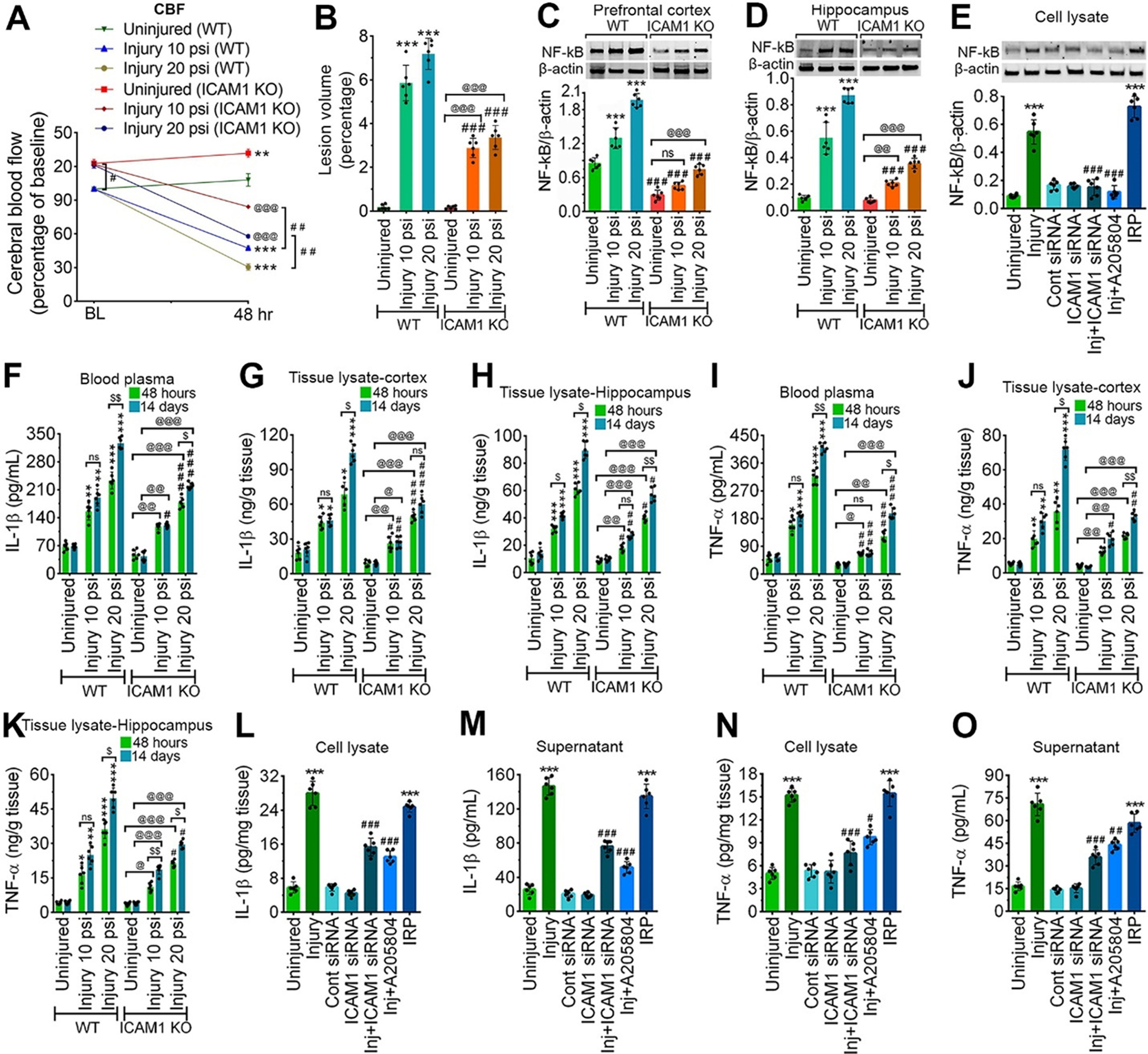
ICAM-1 decreases CBF, increases lesion volume, and activates neuroinflammatory response following both *in vivo* and *in vitro* model of TBI. ***A***, CBF was measured by LDF perfusion monitored at baseline and 48 h after 10 and 20 psi FPI injury in WT and *ICAM-1*^−/−^ animals and compared with their respective baseline values (*n* = 6/group). ***B***, Measurement of lesion volume by cresyl violet staining method (*n* = 6/group). **C-*E*,** Western blotting of NF-kB and β-actin in PFC (***C***) and hippocampal (***D***) tissue lysates 48 h after 10 and 20 psi FPI (*n* = 6/group) and in the cell lysates of hBMVEC (***E***; *n* = 6/group) 24 h after 3.0 psi stretch injury. Bar graphs show the quantification of NF-kB. ***F–H***, ELISA of IL-1β in the blood plasma samples (***F***), the brain prefrontal cortical tissue lysates (***G***) and in the hippocampal tissue lysates (***H***; *n* = 6/group). ***I–K*,** ELISA of TNF-α in the in the blood plasma samples (***I***), the brain prefrontal cortical tissue lysates (***J***) and in the hippocampal tissue lysates (***K***; *n* = 6/group). ***L***, ***M***, ELISA of IL-1β in the cell lysates (***L***) and cell culture supernatant (***M***) of hBMVEC 24 h after 3.0 psi stretch injury (*n* = 6/group). ***N***, ***O***, ELISA of TNF-α in the cell lysates (***N***) and cell culture supernatant (***O***) of hBMVEC (*n* = 6/group) 24 h after 3.0 psi stretch injury. All values are expressed as mean ± SD one-way ANOVA for ***E***, ***L–O*** and two-way ANOVA for ***A–D***, ***F–K*** followed by Bonferroni *post hoc* tests. Statistically significant *p < 0.05, **p < 0.01, ***p < 0.001 versus WT uninjured group; #*p* < 0.05, ##*p* < 0.01, ###*p* < 0.001 versus corresponding WT injury groups in ***A–D*, *F–K***; @*p* < 0.05; @@*p* < 0.01, @@@*p* < 0.001 versus uninjured *ICAM-1*^−/−^ group in ***A–D*, *F–K***; #*p* < 0.05, ##*p* < 0.01, ##*p* < 0.01 between WT and KO groups in their respective groups in ***A–D*, *F–K*.** $*p* < 0.05, $$*p* < 0.01 between 48 h and 14 d postinjury in (***F–K***); ***p < 0.001 versus uninjured hBMVEC in ***E***, ***L–O***; #*p* < 0.05, ##*p* < 0.01, ###*p* < 0.001 versus 3 psi injury in ***E***, ***L–O***; ns = non-significant.

Next, to validate an early event in the pathotrajectory of TBI, we monitored the CBF in the pericontusional region at baseline and 48 h after experimental FPI. Figure 4*A* shows the quantification of CBF monitored by Laser Doppler perfusion method for 1 min taken at baseline and 48 h in uninjured WT, 10 psi, and 20 psi FPI. Quantification of blood flow shows a 52.7% decrease in CBF after 10 psi injury compared with the baseline level. In the 20 psi injury group, we observed a 69.4% decrease in CBF as compared with the baseline level. In WT uninjured group, the CBF observed at 48 h was of similar magnitude with no significant differences when compared with the baseline level (Fig. 4*A*). Similarly, *ICAM-1*^−/−^ animals showed significantly lower CBF in 10 psi (31.02% decrease) and 20 psi (52.59% decrease) compared with the baseline level (*F*_(5,30)_ = 95.32, *p* < 0.0001; Fig. 4*A*). Interestingly, when compared with WT animals, significantly higher CBF was observed in *ICAM-1*^−/−^ animals in uninjured, 10 and 20 psi groups that validate the role of ICAM-1 in CBF (*F*_(2,30)_ = 5.963, *p* = 0.0066; Fig. 4*A*), which are in accord with the previous report shown by Connolly et al. (1996).

### ICAM-1 increases lesion volume and activates neuroinflammatory response following both *in vivo* and *in vitro* model of TBI

Next, we measured the lesion volume by cresyl violet staining as previously reported (Villapol et al., 2015). We observed that 10 and 20 psi injury resulted in a significant increase in the lesion volume in the injury area of both WT and *ICAM-1*^−/−^ mice (*p* < 0.001) compared with their corresponding uninjured groups (Fig. 4*B*). However, when WT and *ICAM-1*^−/−^ groups were compared a significant decrease (*p* < 0.001) in the lesion volume was observed in *ICAM-1*^−/−^ groups compared with the corresponding WT injured groups (Fig. 4*B*).

To examine whether ICAM-1 upregulation after TBI onset regulates proinflammatory response, we analyzed the proinflammatory markers such as NF-kB, IL-1β, and TNF-α in both *in vivo* FPI and *in vitro* stretch injury model. Western blotting revealed that induction of injury (10 and 20 psi) in WT group causes a significant increase in NF-kB expression in both the PFC and hippocampus. In contrast, although ICAM-1 deletion in 20 psi FPI group results in a significant increase in NF-kB in both PFC (*F*_(2,30)_ = 24.67, *p* < 0.0001) and hippocampus (*F*_(2,30)_ = 53.8, *p* < 0.0001) compared with *ICAM-1*^−/−^ uninjured group, we observed a significant decrease in the expression of NF-kB in both 10 and 20 psi injured *ICAM-1*^−/−^ groups compared with the corresponding WT groups (Fig. 4*C*,*D*). Similarly, 3 psi stretch injury in *in vitro* resulted in a 5.99-fold increase in NF-kB protein expression than uninjured control. Interestingly, inhibiting cells with either ICAM-1 siRNA or A205804 significantly decreased the expression level of NF-kB; however, when the cells were induced with IRP, we observed a sharp increase in NF-kB expression (*F*_(6,35)_ = 128.3; *p* < 0.0001; Fig. 4*E*).

Next, evaluation of IL-1β levels using ELISA after 10 and 20 psi FPI animals revealed a substantial increase in IL-1β levels in blood plasma (*F*_(5,30)_ = 33.94, *p* < 0.0001; Fig. 4*F*) and the PFC (*F*_(5,60)_ = 15.6, *p* < 0.0001) and hippocampus (*F*_(5,60)_ = 11.48, *p* < 0.0001) tissue lysates samples of WT groups (Fig. 4*G*,*H*). Although a similar trend of results was observed in *ICAM-1*^−/−^ groups, but values are significantly less compared with the WT group. To further evaluate neuroinflammatory response in the chronic phase of an injury, we compared the levels of IL-1β at day 14 with respect to 48 h postinjury. In the WT 10 psi group, except the hippocampus, we did not observe any significant increase in the level of IL-1β at 14 d when compared with 48 h. However, with moderate injury (20 psi), a marked increase in IL-1β was evident in the blood plasma, PFC, and hippocampus at day 14 in comparison to 48 h. Although, 14 d postinjury resulted in a similar trend in the increase of IL-1β levels in the blood plasma and hippocampus of ICAM-1 deleted 20 psi FPI group in comparison to 48 h, a significant reduction in the levels of IL-1β was evident in both 10 and 20 psi FPI *ICAM-1*^−/−^ when compared with corresponding WT injured groups (Fig. 4*F–H*).

Next, to determine whether ICAM-1 deletion downregulates TNF-α, we quantified the levels of TNF-α over time (48 h and 14 d). Upregulation of TNF-α, as measured by ELISA was evident in the blood plasma (*F*_(5,30)_ = 55.11, *p* < 0.0001), PFC (*F*_(5,30)_ = 51.98, *p* < 0.0001), and hippocampus (*F*_(5,60)_ = 11.48, *p* < 0.0001) of both 10 and 20 psi WT and *ICAM-1*^−/−^ mice compared with their corresponding WT uninjured group (Fig. 4*I–K*). ICAM-1 deletion resulted in significantly reduced levels of TNF-α in the blood plasma, PFC, and hippocampus of both 10 and 20 psi injured groups compared with the corresponding WT injured groups (Fig. 4*I–K*). These results suggest that while a significant elevation in the levels of TNF-α occurs following TBI, levels of biologically active TNF-α are significantly reduced concurrent depletion of ICAM-1. Similar to IL-1β, the temporal changes (48 h vs 14 d) in the levels of TNF-α were observed in the blood plasma, PFC, and hippocampus of both WT and *ICAM-1*^−/−^ groups with 20 psi FPI injury. (Fig. 4*I–K*). Similarly, 3 psi stretch injury caused a significant release of IL-1β in the cell lysate (*F*_(6,35)_ = 198.2, *p* < 0.0001) and cell supernatant (*F*_(6,35)_ = 262.1, *p* < 0.0001). TNF-α levels in both cell lysate (*F*_(6,35)_ = 82.84, *p* < 0.0001) and cell supernatant (*F*_(6,35)_ = 145.8, *p* < 0.0001) were also significantly increased with stretch injury, however, the levels of both IL-1β and TNF-α were significantly reduced on prior treatment of the cells with either ICAM-1 siRNA or A205804. As expected, the induction of IRP leads to a substantial increase and release of IL-1β and TNF-α in both cell lysate and cell supernatant (Fig. 4*L–O*).

### TBI-induced ICAM-1 triggers the activation of caspase-3 *in vivo* and *in vitro*

**Figure 5.**
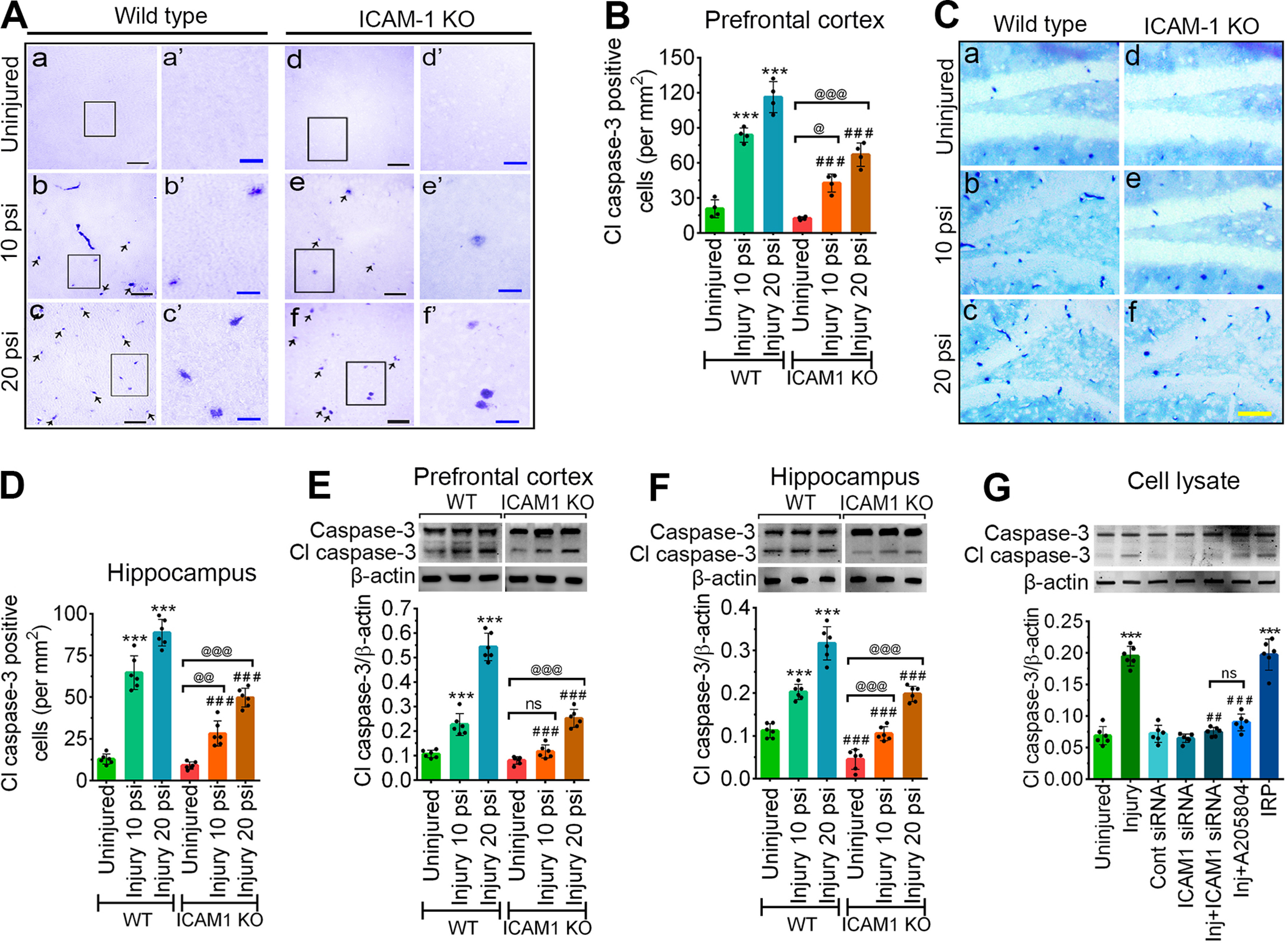
ICAM-1 activates the caspase-3 enzyme. ***A–C***, AP immunohistochemistry of cl-caspase-3 in the PFC (***A***) and hippocampus (***C***) tissue sections from WT and *ICAM-1*^−/−^ mice 48 h after 10 and 20 psi FPI using vector purple substrate kit, SK4600 in ***A*** and vector blue substrate kit, SK5300 in ***C***. Scale bar: 100 μm (***A***, black), 20 μm (***A***, blue), and 100 μm (***C***, yellow). ***B***, ***D***, Quantification of cl-caspase-3-positive cells expressed as per mm^2^ area of sections in WT and *ICAM-1*^−/−^ in uninjured, 10 and 20 psi groups in the PFC (***B***) and hippocampus (***D***) 48 h following FPI (*n* = 6/group). ***E***, ***F***, Western blotting of cl-caspase-3 and β-actin in the PFC (***E***) and hippocampus (***F***) tissue lysates of WT and *ICAM-1*^−/−^ mice 48 h after 10 and 20 psi FPI. The bar graph shows the quantification of cl-caspase-3 versus β-actin (*n* = 6/group). ***G***, Western blotting of cl-caspase-3 and β-actin 24 h after 3.0 psi stretch injury in the cell lysates of hBMVEC treated with control siRNA, ICAM-1 siRNA, A205804, and IRP (*n* = 6/group). Bar graph represents the densitometric ratio of cl-caspase-3 bands versus β-actin bands. All values are expressed as mean ± SD one-way ANOVA for ***G*** and two-way ANOVA for ***B***, ***D–F*** followed by Bonferroni *post hoc* tests. Statistically significant ***p < 0.001 versus WT uninjured group in ***B***, ***D***, ***E***, ***F***; @*p* < 0.05; @@*p* < 0.01, @@@*p* < 0.001 versus uninjured *ICAM-1*^−/−^ group in ***B***, ***D***, ***E***, ***F***; ###*p* < 0.001 versus corresponding WT uninjured or injury groups (10 and 20 psi) in ***B***, ***D***, ***E***, ***F***; ***p < 0.001 versus uninjured hBMVEC in ***G***; #*p* < 0.05, ###*p* < 0.001 versus 3 psi injury in ***G***; ns = non-significant.

To investigate whether activation of ICAM-1 induces apoptosis cascade, we analyzed the expression of the cleaved form of caspase-3 (cl-caspase-3), an apoptosis-inducing enzyme. In AP immunostaining, we observed that 10 and 20 psi injury results in a significant increase in the number of cl-caspase-3-positive cells in the PFC (*F*_(2,18)_ = 12.96, *p* = 0.0003) and hippocampus (*F*_(2,30)_ = 25.71, *p* < 0.0001) of both WT and *ICAM-1*^−/−^ mice compared with their corresponding uninjured groups. However, when WT and *ICAM-1*^−/−^ groups were compared a significant decrease in the number of cl-caspase-3-positive cells was observed in *ICAM-1*^−/−^ groups compared with the corresponding WT injured groups (Fig. 5*A–D*). Western blotting analysis further revealed a significant increase in the expression level of cl-caspase-3 in both 10 and 20 psi WT injury group compared with the uninjured WT control. Although in *ICAM-1*^−/−^ mice, both PFC (*F*_(2,30)_ = 43.48, *p* < 0.0001) and hippocampus (*F*_(2,30)_ = 3.697, *p* = 0.0367) show a slight increase in cl-caspase-3 expression in the injured groups (10 and 20 psi), a significant reduction in cl-caspase-3 expression was observed compared with the corresponding WT injury groups indicating that *ICAM-1*^−/−^ significantly attenuates the induction of apoptosis cascade (Fig. 5*E*,*F*). Similarly, *in vitro* stretch injury at 3 psi in hBMVEC resulted in a 2.82-fold increase in the expression of cl-caspase-3 compared with uninjured control cells. Interestingly, compared with 3 psi stretch injury, induction of ICAM-1 siRNA, A205804 (ICAM-1 inhibitor) before injury causes a significant decrease in the expression level of cl-caspase-3. As expected, the induction of IRP leads to a substantial increase in cl-caspase-3 similar to the levels as reported in the injury group alone (*F*_(6,35)_ = 99.66, *p* < 0.0001; Fig. 5*G*).

### ICAM-1 activation induces apoptosis in animal and cell models of TBI

**Figure 6.**
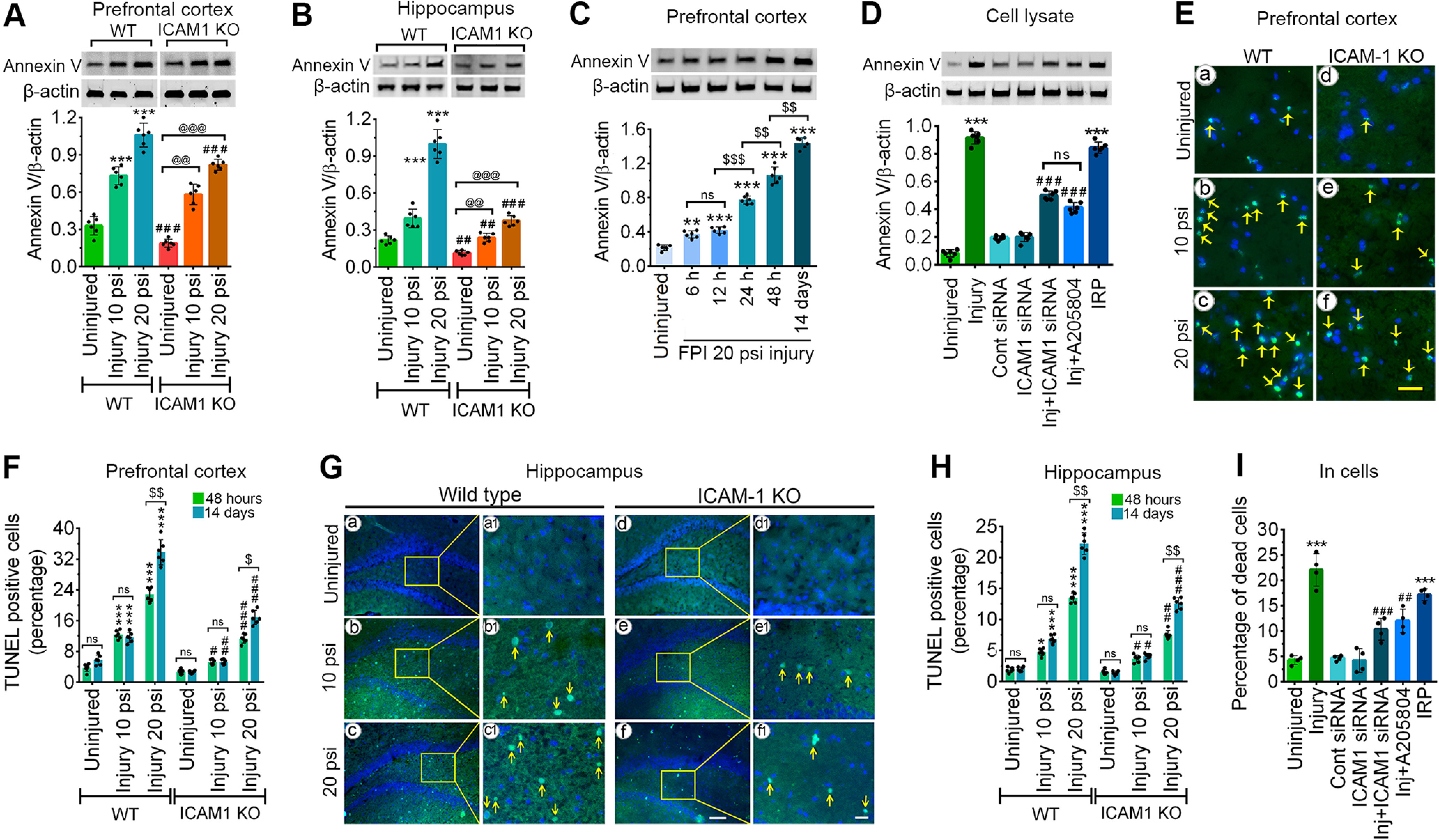
ICAM-1 activation induces cell death in animal and cell models of TBI. ***A***, ***B***, Western blotting of Annexin V and β-actin in tissue lysates of PFC (***A***), hippocampus (***B***) of WT and *ICAM-1*^−/−^ mice 48 h after 10 and 20 psi FPI. ***C***, Western blotting of Annexin V and β-actin at different time points in the PFC 48 h after injury. ***D***, Western blotting of Annexin V and β-actin in the cell lysates of hBMVEC 24 h after 3.0 psi stretch injury. Bar graph represents the densitometric ratio of cl-caspase-3 bands versus β-actin bands (*n* = 6/group). ***E***, ***G***, Representative TUNEL staining (green) images 48 h after 10 and 20 psi FPI in the PFC (***E***) and hippocampus (***G***). Scale bar: 25 μm (***E***), 100 μm (***G***, bigger panels ***a–f***), and 20 μm (***G***, enlarged panels ***a1–f1***); *n* = 6/group. ***F***, ***H***, Percentage of apoptotic-positive cells in the PFC (***F***) and hippocampus (***H***). ***I***, Trypan blue staining represented as a percentage of dead cell analyzed 24 h after 3.0 psi stretch injury in hBMVEC (*n* = 4/group). All values are expressed as mean ± SD one-way ANOVA for ***C***, ***D***, one-way and two-way ANOVA for ***A***, ***B***, ***F***, ***H*** followed by Bonferroni *post hoc* tests. Statistically significant *p < 0.05, **p < 0.01, ***p < 0.001 versus WT uninjured group in ***B–D***, ***F–I*** and versus uninjured hBMVEC in ***E***, ***J***; #*p* < 0.05, ##*p* < 0.01, ###*p* < 0.001 versus corresponding WT injury groups in ***B–D***, ***F–I*** and versus injured hBMVEC in ***E***, ***J***; @*p* < 0.05, @@*p* < 0.01, @@@*p* < 0.001 versus uninjured *ICAM-1*^−/−^ group in ***B–D***, ***F–I***; $*p* < 0.05, $$*p* < 0.01 between 48 h and 14 d postinjury in ***G***, ***I*** and between two time frames in ***D***; ns = non-significant.

Next, we assessed the neurodegeneration in the cortex and hippocampus of the ipsilateral side of the brain. Assessment of apoptosis in the PFC (*F*_(2,3)_ = 14.09, *p* = 0.0298) and hippocampus (*F*_(2,30)_ = 61.88, *p* < 0.0001) using western blotting confirms marked elevation in Annexin V protein expression levels in injury-induced groups (10 and 20 psi FPI) compared with their corresponding controls (Fig. 6*A*,*B*). Comparison of WT and *ICAM-1*^−/−^ mice reflects that deletion of ICAM-1 greatly attenuated the expression of Annexin V thus validating the regulatory role of ICAM-1 in inducing apoptosis. The temporal expression of Annexin V in the PFC at various times following 20 psi FPI displayed a significant increase in the expression of Annexin V protein level at 6 h, 12 h, 24 h, 48 h, and 14 d consistent with a similar trend in the increase of ICAM-1 as observed in Figure 1*K* thus establishing the correlation that exists between ICAM-1 and apoptosis (*F*_(5,30)_ = 398.7, *p* < 0.0001; Fig. 6*C*). Similarly, 3 psi FPI stretch injury in hBMVEC resulted in a marked increase in the expression of Annexin V compared with uninjured control cells. Although treatment of IRP alone shows a similar level of expression of Annexin V as observed in injured groups, induction of ICAM-1 siRNA, A205804 (ICAM-1 inhibitor) before injury significantly reduced the expression level of Annexin V (*F*_(6,35)_ = 564.4, *p* < 0.0001; Fig. 6*D*).

TUNEL staining further revealed that in both PFC and hippocampus, a significant increase in positive apoptotic cells (green color) was observed when compared with their corresponding uninjured groups. A significant temporal increase in the positive apoptotic cells was evident only within WT or *ICAM-1*^−/−^ 20 psi groups when 48 h and 14 d were compared (PFC: *F*_(5,30)_ = 57.84, *p* < 0.0001; hippocampus: *F*_(5,30)_ = 68.78, *p* < 0.0001). Comparison of WT and *ICAM-1*^−/−^ mice reflects that deletion of ICAM-1 greatly attenuated the number of apoptotic-positive cells in both 10 and 20 psi FPI groups (Fig. 6*E–H*). Since we observe the structural changes in the endothelial cell culture monolayer and the shape of the cells in 3 psi injury inflicted cells (data not shown), we further assessed ICAM-1 induced cell death using trypan blue staining. The result shows that although 3 psi stretch injury or injury-induced by IRP causes an increased percentage of dead cells, pretreatment of ICAM-1 siRNA or A205804 significantly attenuated the number of dead cells (*F*_(6,21)_ = 48.24, *p* < 0.0001; Fig. 6*I*).

### ICAM-1 causes sensorimotor deficits and psychological stress following TBI

**Figure 7.**
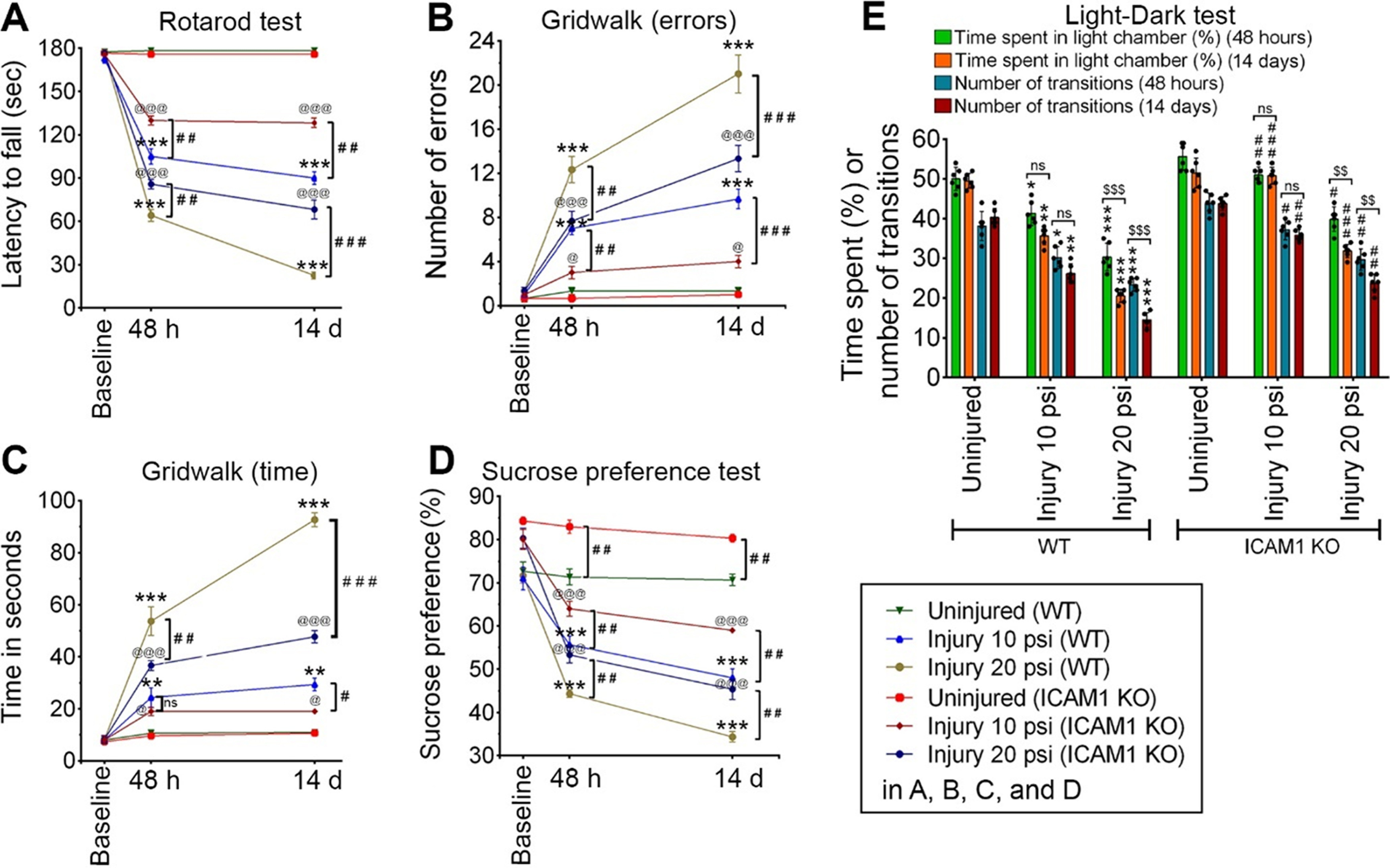
ICAM-1 activation impairs sensorimotor functions and enhances psychological stress after TBI. ***A***, Latency to fall time in rotarod was examined in WT and *ICAM-1*^−/−^ mice at baseline, 48 h and 14 d after 10 and 20 psi FPI (*n* = 6/group). ***B***, ***C***, The grid-walk analysis was monitored in WT and *ICAM-1*^−/−^ mice at baseline, 48 h and 14 d after 10 and 20 psi FPI. Number of grid-walk errors (***B***) and time to finish a grid-walk (***C***) (*n* = 6/group). ***D***, The sucrose preference test for depression behavior was calculated as a percentage of the volume of sucrose intake over the total volume of fluid intake at baseline, 48 h, and 14 d after injury in WT and *ICAM-1*^−/−^ mice subjected to 10 and 20 psi FPI (*n* = 6/group). ***E***, The light-dark box test for anxiety-like behavior expressed as % time spent exploring the light chamber and the number of transitions between the chambers monitored at 48 h and 14 d after injury in WT and *ICAM-1*^−/−^ mice subjected to 10 and 20 psi FPI (*n* = 6/group). All values are expressed as mean ± SD two-way ANOVA followed by Bonferroni *post hoc* tests. Statistically significant *p < 0.05, **p < 0.01, ***p < 0.001 versus WT uninjured group; @*p* < 0.05; @@*p* < 0.01, @@@*p* < 0.001 versus *ICAM-1*^−/−^ uninjured group; #*p* < 0.05, ##*p* < 0.01, ###*p* < 0.001 versus corresponding WT injury groups; $$*p* < 0.01 between 48 h and 14 d postinjury in ***A–E***; ns = non-significant.

We next examined whether ICAM-1 deletion has a significant role in the recovery of functional outcome in *in vivo* model of TBI. Assessment of sensorimotor function confirms that deletion of ICAM-1 shows a substantial increase in time for latency to fall in the rotarod as compared with WT 10 and 20 psi injured animals at 48 h and 14 d as observed in a rotarod test (*F*_(10,60)_ = 106.1, *p* < 0.0001; Fig. 7*A*). Grid-walk test as a measure of sensorimotor function further validates that compared with WT injured mice, ICAM-1 deletion in both 10 and 20 psi FPI mice significantly reduces the number of errors (*F*_(10,24)_ = 39.92, *p* < 0.0001) and time (*F*_(10,24)_ = 85.98, *p* < 0.0001) to complete one grid walk length (Fig. 7*B*,*C*). Assessment of psychological stress behavior such as depression and anxiety were measured using the sucrose preference test and light-dark box test, respectively. In the sucrose preference test, although both WT and *ICAM-1*^−/−^ 10 and 20 psi groups showed a significant reduction in the sucrose preference with respect to the corresponding uninjured groups at 48 h and 14 d post-TBI (*F*_(10,24)_ = 28.34, *p* < 0.0001; Fig. 7*D*), ICAM-1 deletion substantially improves sucrose preference at 48 h and 14 d when compared with WT 10 and 20 psi injured animals (Fig. 7*D*). In the light-dark box test, ICAM-1 deletion monitored at 48 h and 14 d postinjury in both 10 and 20 psi injury group causes a significant increase in latency to stay in the light chamber or number of transition when compared with WT 10 and 20 psi injured mice (*F*_(15,48)_ = 10.25, *p* < 0.0001; Fig. 7*E*), indicating that ICAM-1 is partly involved in regulating depression and anxiety-like psychological stress behavior.

**Figure 8.**
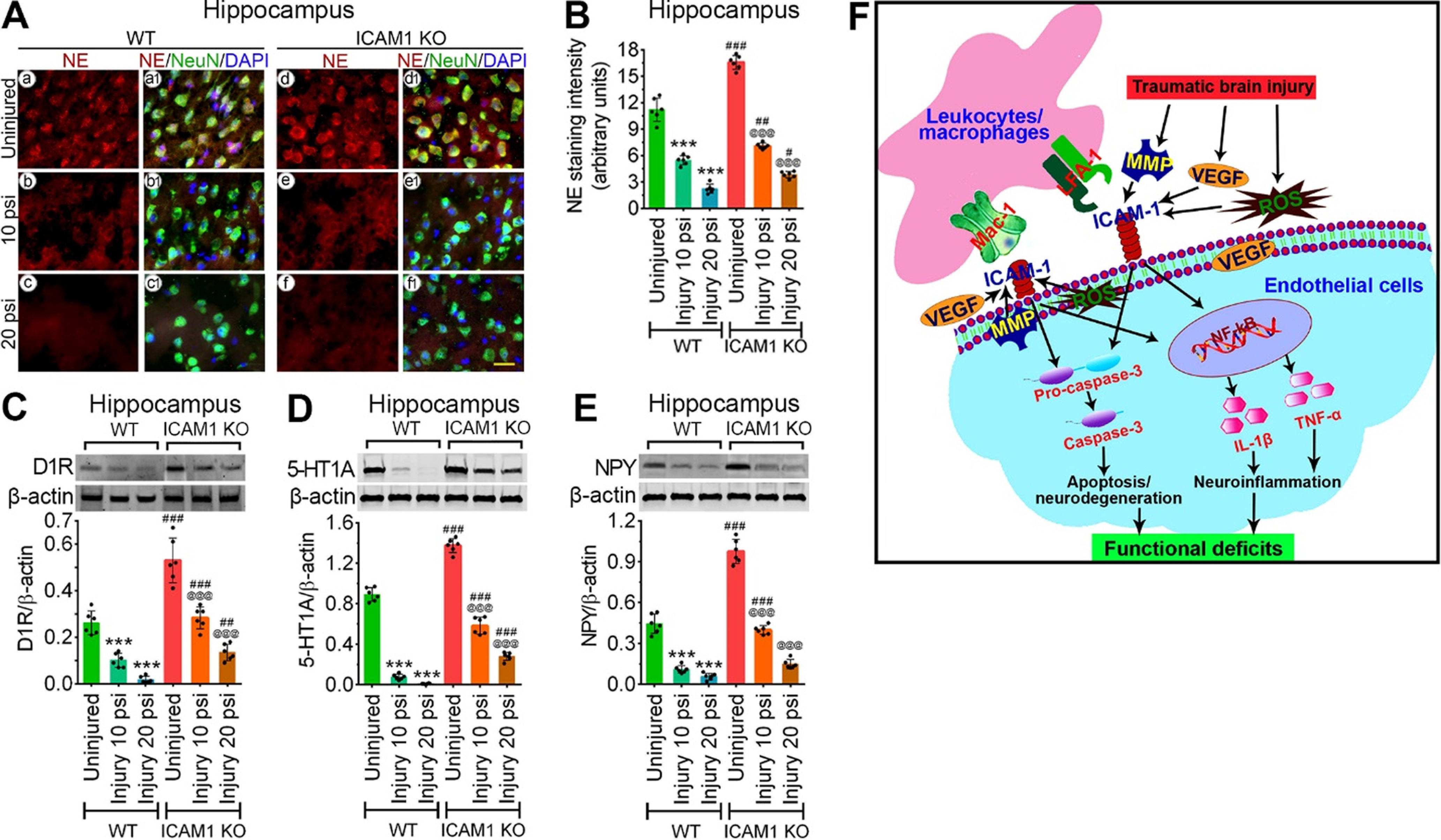
ICAM-1 reduces neurotransmitters expression that reflects in sensorimotor deficits and psychological stress after TBI. ***A***, Immunofluorescent staining of NE (red) in the hippocampus area of WT and *ICAM-1*^−/−^ mice after 10 and 20 psi FPI and merged with NeuN (green) and DAPI (blue). Scale bar: 20 μm (all panels). ***B***, Quantification of NE staining in the hippocampus area of uninjured, 10 and 20 psi FPI WT and *ICAM-1*^−/−^ mice using ImageJ software (*n* = 6/group). ***C–E***, Western blot analysis of 5-HT1AR (***C***), DAD1R (***D***), NPY (***E***) and β-actin in the tissue lysates of hippocampus of WT and *ICAM-1*^−/−^ mice 48 h after 10 and 20 psi FPI. The bar graph with dot plots shows the quantification of 5-HT1AR (***C***), DAD1R (***D***), NPY (***E***) versus β-actin (*n* = 6/group). ***F***, Schematic presentation of the findings. All values are expressed as mean ± SD two-way ANOVA followed by Bonferroni *post hoc* tests. Statistically significant ***p < 0.001 versus WT uninjured group; ^@@@^*p* < 0.001 versus uninjured *ICAM-1*^−/−^ group; #*p* < 0.05, ##*p* < 0.01, ###*p* < 0.001 versus WT corresponding injury groups; ns = non-significant. NE, norepinephrine; 5-HT1AR, 5-HT 1A receptor; DAD1R, DA D1 receptor; NPY, neuropeptide Y.

To evaluate the link between impairment of functional outcome with dysregulation in the neurotransmitter signaling pathways (Geyer et al., 2001; Wurm et al., 2007; Aan Het Rot et al., 2009; Mickey et al., 2011; Hamon and Blier, 2013), we next demonstrated the role of ICAM-1 in regulating neurotransmitters expression and thereby control sensorimotor function, and depression and anxiety-like psychological stress behaviors. In immunofluorescence staining just below the injury area in the CA1 region of the hippocampus, 10 and 20 psi FPI caused a significant decrease in the expression of NE in WT and *ICAM-1*^−/−^ mice compared with their corresponding uninjured groups. However, ICAM-1 deletion markedly attenuated the decrease in the expression of NE (*F*_(2,30)_ = 26.88, *p* < 0.0001) compared with WT injury groups (Fig. 8*A*,*B*). Both WT and ICAM-1 KO injury groups also showed a similar significant decrease in the expression levels of the DAD1R (*F*_(2,30)_ = 6.418, *p* = 0.0048), 5-HT1AR (*F*_(2,30)_ = 16.55, *p* < 0.0001), and NPY (*F*_(2,30)_ = 53.16, *p* < 0.0001) when compared with their respective uninjured groups. Although, induction of TBI was sufficient to attenuate the expression levels of DAD1R, 5-HT1AR and NPY in both WT and ICAM-1 animals, ICAM-1 deletion had a significant role in restoring the expression levels of DAD1R, 5-HT1AR and NPY when compared with the WT 10 and 20 psi injured mice (Fig. 8*C–E*).

## Discussion

ICAM-1 plays a major role in many facets in the pathogenesis of TBI (Yang et al., 2005; Kumar et al., 2017). However, the underlying mechanisms that drive the regulatory role of ICAM-1 in pathogenesis and subsequent psychological stress associated with TBI are largely unknown. In the present study, using the FPI model in mice and the stretch injury model in hBMVEC, we demonstrated the downstream mechanisms of ICAM-1 in TBI-induced neuroinflammatory cascades and neurodegeneration. We have further validated our results *in vivo* using *ICAM-1^−/−^* mice, and *in vitro* by knocking down ICAM-1 gene using ICAM-1 siRNA and pharmacologically inhibiting ICAM-1. Stretch injury model in hBMVEC demonstrated the mechanisms of the upstream processes that possibly trigger the induction of ICAM-1. Further, we analyzed the link between ICAM-1 induced neuroinflammation and neurodegeneration with sensorimotor function impairment and depression and anxiety-like psychological stress behavior. Figure 8*F* depicts a schematic presentation of our findings.

ICAM-1 is a cell surface glycoprotein, expressed on endothelial cells and cells of the immune system (de Fougerolles et al., 1994) and regulates leukocyte-endothelial transmigration via interaction with a group of glycoproteins such as leukocyte function-associated antigen (LFA-1) on leukocytes and Mac-1 on neutrophils, monocytes, and macrophages (Henderson et al., 2001; Romanova and Mushinski, 2011). Over-expression of ICAM-1 triggers other cellular signaling pathways that contribute to the pathophysiology of various diseases such as TBI (Rancan et al., 2001; Hamaï et al., 2008) that leads to inflammation and cell death (Frank and Lisanti, 2008). Our *in vivo* and *in vitro* results are in good accord with the previous report that validates the activation of ICAM-1 and its receptors LFA-1 and Mac-1 following TBI (Frank and Lisanti, 2008). We further linked the induction of ICAM-1 to the activation of NF-kB and the cytokines such as IL-1β and TNF-α *in vitro* and *in vivo* models of TBI. We used both mild (10 psi) and moderate (20 psi) injury to monitor the levels of cytokines at 48 h and 14 d. Although our data revealed that temporal increase in the levels of IL-1β and TNF-α were not significantly increased with mild injury (10 psi) measured at 48 h and 14 d, a significant increase was evident with moderate injury (20 psi). Since the extent of injury in moderate group (20 psi) is high, the innate ability to restore the levels of cytokines even after 14 d is compromised as compared with mild injury group. Moreover, similar temporal studies to access the levels of IL-1β and TNF-α following injury are in well accord to our findings that shows increased levels of cytokine release in the serum sample following TBI (PMID 31708858). Previously, we reported that TBI induces neuroinflammation, activation of MMPs, and BBB disruption via various signaling cascades in different models of TBI (Abdul-Muneer et al., 2013, 2015, 2018; Bhowmick et al., 2018, 2019a,b). In this study, the role of oxidative stress in activating ICAM-1 is evident in our data since NOX inhibitor; apocynin significantly reduced the level of ICAM-1. Several investigators have reported that TBI induces oxidative stress and stimulates the production of ROS by upregulating NADPH oxidases (Landmesser et al., 2007). This study showed that oxidative stress is one of the factors that activate ICAM-1, which in turn causes neuroinflammation and neurodegeneration following TBI.

Our recent work showed that oxidative stress induces neuroinflammation, neurodegeneration, BBB damage, and infiltration of immune cells to the perivascular region in different models of mTBI (Abdul-Muneer et al., 2013, 2017b, 2018; Patel et al., 2017), which raise the possibility of a bidirectional relationship that exists between oxidative stress and inflammation that induces apoptosis, lipid peroxidation, oxidative stress, or free radical formation (Bao et al., 2012; Weaver et al., 2015). In this study, MMP-mediated activation of ICAM-1 was evident with the treatment with MMP inhibitor, TIMP1. MMP activation and neurodegeneration have been associated with several neurologic diseases (Abdul Muneer et al., 2012; Trentini et al., 2015), including TBI (Mannello et al., 2005). Activated MMPs by oxidative stress cleave extracellular matrix and tight junction proteins that impair brain endothelium stability, thereby compromising the BBB (Abdul-Muneer et al., 2013). Several reported studies on the role of VEGF-A in the induction of ICAM-1 in different neurologic disorders led us to analyze the role of VEGF-A and its receptor VEGFR-2 in the induction of ICAM-1 in our *in vitro* model. We demonstrated that the high concentration of VEGF upregulates the expression of ICAM-1 protein. However, VEGF-A is not the actual causative factor for the induction of ICAM-1, but phosphorylation of VEGFR-2 protein induces the expression of ICAM-1. This is evident from the treatment with Ki8751, an inhibitor of VEGFR-2 phosphorylation.

Recent works, both *in vitro* and *in vivo*, indicates that two receptors of the ICAM-1, LFA-1 (CD11a/CD18) and Mac-1 (CD11b/CD18) activates the signaling mechanisms of transmigration of leukocytes (Dustin et al., 1988; Springer, 1990) and may have important and distinct roles in the inflammatory process (Ding et al., 1999; Henderson et al., 2001). Upon activation and strong adherence, the leukocytes flatten and soon undergo diapedesis to enter the parenchyma of the brain that advocates LFA-1/ICAM-1 interaction phenomena as a therapeutic target against various pathophysiology conditions of neurologic disorders, and cancer (Rancan et al., 2001; Hamaï et al., 2008). Our results are in line with previous studies that strongly support that a strong cooperative interaction of ICAM-1 with its receptors LFA-1 and Mac-1 exist following injury and this cooperative interaction apart from leukocyte transmigration plays a major role in downstream neuroinflammatory and pathophysiology outcomes. As observed in the results, both WT and ICAM1 KO animals in uninjured groups displayed a comparable basal level in the expression of LFA-1 and Mac-1. Although we did not observe a significant change in the expression of LFA-1 and Mac-1 in ICAM-1 KO injured groups with respect to uninjured ICAM-1 KO groups, our results strongly suggest that ICAM-1 depletion plays a significant role in regulating the expression of both LFA-1 and Mac-1 since ICAM-1 KO in injury groups (10 and 20 psi) significantly reduced the expression levels of LFA-1 and Mac-1 when compared with their respective WT injury groups. The role of LFA-1 or Mac-1 is evident from the fact that the leukocyte rolling velocity is increased in either *LFA-1*^−/−^ or *Mac-1*^−/−^ mice (KO mice) when compared with the WT (Henderson et al., 2001; Dunne et al., 2003). However, both LFA-1 and Mac-1 appear to be important in leukocyte adhesion, the contribution from LFA-1 is greater than that from Mac-1 (Ding et al., 1999; Dunne et al., 2002). As with the other integrins, LFA-1 does not function as a mere adhesive contact between cells, but it also mediates signals that modulate their growth, differentiation, and survival.

Since a strong connection exists between transmigration of immune cells and neuroinflammation following TBI (Soares et al., 1995), in our study, we linked the activation of ICAM-1 with inflammatory cascades and concluded that ICAM-1 deletion or inhibition attenuates proinflammatory response such as NF-kB, IL-1β, and TNF-α. Although ICAM-1 exerts both direct and indirect effects contributing to neuronal death *in vitro* and *in vivo* (Lindsberg et al., 1996), we showed that ICAM-1 directly induced apoptosis through the activation of caspase-3 since injured cells pretreated with ICAM-1 siRNA and A205804 (ICAM-1 inhibitor) markedly reduced cl-caspase-3 expression. Our TUNEL assay further validates the role of ICAM-1 in cell death. Previous studies have shown that CD11d/CD18 integrins bind to adhesion molecules such as VCAM-1 in a rat model and ICAM-1 in humans infiltrating leukocytes and mediates inflammatory response that leads to worsening of secondary damage (Hogg and Leitinger, 2001). Evidence also shows that blocking CD11d/CD18 integrins using an anti-CD11d monoclonal antibody as a therapeutic strategy to improve neurologic outcomes after TBI or repeated concussion (Bao et al., 2012; Weaver et al., 2015). Based on these studies, it becomes evident that an in-depth understanding of the interaction of ICAM-1 and integrins in highly warranted and similar treatment regimens of inhibiting ICAM-1 and integrins could pose a potential therapeutic strategy in attenuating ICAM-1-mediated neuroinflammation and secondary damage associated with TBI.

TBI is known to produce acute and prolonged sensorimotor impairment and psychological stress behavior associated with inflammatory changes in brain regions (Xing et al., 2013; Bhowmick et al., 2018). ICAM-1 upregulation in the vasculature of brain regions has been previously implicated in fear and anxiety responses (Zhang et al., 2017). Our current study extends this finding to show that TBI-induced ICAM-1 activation is associated with impairment in sensorimotor function and depression and anxiety-like psychological stress disturbances. Our data indicate that ICAM-1 activation following TBI induced a significant impairment in sensorimotor function as monitored by rotarod and grid walk test and severely exacerbate depression and anxiety-like psychological stress as monitored by the sucrose preference test and the light-dark test. Further validation of the role of ICAM-1 in impairment of functional recovery following TBI is supported by the fact that the deletion of ICAM-1 significantly improves functional recovery. The changes in sensorimotor impairment and psychological stress in WT and *ICAM-1^−/−^* mice were in correlation with the expression level of NE, 5-HT1A, D1R, and NPY markers in uninjured, and 10 and 20 psi injured animals since deletion in ICAM-1 in injury groups significantly restored the expression levels of NE, 5-HT1A, D1R, and NPY markers with significant recovery in functional outcomes. Collectively, these findings provide novel evidence for the contribution of adhesion molecules (ICAM-1) in facilitating the impairment in functional outcome in response to TBI.

In conclusion, for the first time, we shed light on the specific role of ICAM-1 in impairing sensorimotor function and enhancing psychological stress by studying the mechanisms of ICAM-1^-^mediated neuroinflammation and neurodegeneration via the pathway of LFA-1 or Mac-1 proteins in hBMVEC stretch injury and FPI animal models. The analysis of the role of ICAM-1 has opened a new therapeutic target that could be used to treat TBI-induced neurologic complications by blocking the activation of ICAM-1, thereby reduce the transmigration of immune cells to the brain, neuroinflammation, and cell death. Our present report encourages further work to undertake a comprehensive analysis to evaluate the therapeutic efficacy of blocking ICAM-1 to alleviate neurovascular damages and functional deficits *in vivo*.

## Synthesis

Reviewing Editor: Erica Glasper, University of Maryland Decisions are customarily a result of the Reviewing Editor and the peer reviewers coming together and discussing their recommendations until a consensus is reached. When revisions are invited, a fact-based synthesis statement explaining their decision and outlining what is needed to prepare a revision will be listed below. Note: If this manuscript was transferred from JNeurosci and a decision was made to accept the manuscript without peer review, a brief statement to this effect will instead be what is listed below.

This manuscript was transferred from the Journal of Neuroscience. The authors have sufficiently revised this manuscript based on the thorough comments from the prior reviews. The manuscript is acceptable for publication in eNeuro.

## References

[B1] Aan Het Rot M, Mathew SJ, Charney DS (2009) Neurobiological mechanisms in major depressive disorder. CMAJ 180:305–313. 10.1503/cmaj.080697 19188629 PMC2630359

[B2] Abdul Muneer PM, Alikunju S, Szlachetka AM, Murrin LC, Haorah J (2011) Impairment of brain endothelial glucose transporter by methamphetamine causes blood-brain barrier dysfunction. Mol Neurodegener 6:23. 10.1186/1750-1326-6-23 21426580 PMC3073895

[B3] Abdul Muneer PM, Alikunju S, Szlachetka AM, Haorah J (2012) The mechanisms of cerebral vascular dysfunction and neuroinflammation by MMP-mediated degradation of VEGFR-2 in alcohol ingestion. Arterioscler Thromb Vasc Biol 32:1167–1177. 10.1161/ATVBAHA.112.247668 22402362 PMC3501346

[B4] Abdul-Muneer PM, Schuetz H, Wang F, Skotak M, Jones J, Gorantla S, Zimmerman MC, Chandra N, Haorah J (2013) Induction of oxidative and nitrosative damage leads to cerebrovascular inflammation in an animal model of mild traumatic brain injury induced by primary blast. Free Radic Biol Med 60:282–291. 10.1016/j.freeradbiomed.2013.02.029 23466554 PMC4007171

[B5] Abdul-Muneer PM, Chandra N, Haorah J (2015) Interactions of oxidative stress and neurovascular inflammation in the pathogenesis of traumatic brain injury. Mol Neurobiol 51:966–979. 10.1007/s12035-014-8752-3 24865512 PMC9420084

[B6] Abdul-Muneer PM, Conte AA, Haldar D, Long M, Patel RK, Santhakumar V, Overall CM, Pfister BJ (2017a) Traumatic brain injury induced matrix metalloproteinase2 cleaves CXCL12α (stromal cell derived factor 1α) and causes neurodegeneration. Brain Behav Immun 59:190–199. 10.1016/j.bbi.2016.09.002 27614125

[B7] Abdul-Muneer PM, Alikunju S, Mishra V, Schuetz H, Szlachetka AM, Burnham EL, Haorah J (2017b) Activation of NLRP3 inflammasome by cholesterol crystals in alcohol consumption induces atherosclerotic lesions. Brain Behav Immun 62:291–305. 10.1016/j.bbi.2017.02.014 28232172 PMC6378699

[B8] Abdul-Muneer PM, Bhowmick S, Briski N (2018) Angiotensin II causes neuronal damage in stretch-injured neurons: protective effects of losartan, an angiotensin T1 receptor blocker. Mol Neurobiol 55:5901–5912. 10.1007/s12035-017-0812-z 29119534

[B9] Amunts K, Kedo O, Kindler M, Pieperhoff P, Mohlberg H, Shah NJ, Habel U, Schneider F, Zilles K (2005) Cytoarchitectonic mapping of the human amygdala, hippocampal region and entorhinal cortex: intersubject variability and probability maps. Anat Embryol (Berl) 210:343–352. 10.1007/s00429-005-0025-5 16208455

[B10] Bankhead P (2014) Analyzing fluorescence microscopy images with ImageJ. Belfast: Queen’s University Belfast.

[B11] Bao F, Shultz SR, Hepburn JD, Omana V, Weaver LC, Cain DP, Brown A (2012) A CD11d monoclonal antibody treatment reduces tissue injury and improves neurological outcome after fluid percussion brain injury in rats. J Neurotrauma 29:2375–2392. 10.1089/neu.2012.2408 22676851 PMC4854615

[B12] Bevilacqua MP (1993) Endothelial-leukocyte adhesion molecules. Annu Rev Immunol 11:767–804. 10.1146/annurev.iy.11.040193.004003 8476577

[B13] Bhowmick S, D’Mello V, Ponery N, Abdul-Muneer PM (2018) Neurodegeneration and sensorimotor deficits in the mouse model of traumatic brain injury. Brain Sci 8:11. 10.3390/brainsci8010011 PMC5789342 29316623

[B14] Bhowmick S, D’Mello V, Caruso D, Wallerstein A, Abdul-Muneer PM (2019a) Impairment of pericyte-endothelium crosstalk leads to blood-brain barrier dysfunction following traumatic brain injury. Exp Neurol 317:260–270. 10.1016/j.expneurol.2019.03.014 30926390

[B15] Bhowmick S, D’Mello V, Caruso D, Abdul-Muneer PM (2019b) Traumatic brain injury-induced down regulation of Nrf2 activates inflammatory response and apoptotic cell death. J Mol Med (Berl) 97:1627–1641. 10.1007/s00109-019-01851-4 31758217

[B16] Boccella S, Iannotta M, Cristiano C, Iannotti FA, Bello FD, Guida F, Belardo C, Infantino R, Ricciardi F, Giannella M, Calignano A, Di Marzo V, Maione S, Luongo L (2020) Treatment with 2-pentadecyl-2-oxazoline restores mild traumatic brain injury-induced sensorial and neuropsychiatric dysfunctions. Front Pharmacol 11:91. 10.3389/fphar.2020.00091 32161542 PMC7052365

[B17] Bourin M, Hascoët M (2003) The mouse light/dark box test. Eur J Pharmacol 463:55–65. 10.1016/S0014-2999(03)01274-3 12600702

[B18] Brigitta B (2002) Pathophysiology of depression and mechanisms of treatment. Dialogues Clin Neurosci 4:7–20. 22033824 10.31887/DCNS.2002.4.1/bbondy PMC3181668

[B20] Cole SW (2008) Social regulation of leukocyte homeostasis: the role of glucocorticoid sensitivity. Brain Behav Immun 22:1049–1055. 10.1016/j.bbi.2008.02.006 18394861 PMC3004947

[B21] Connolly ES Jr, Winfree CJ, Springer TA, Naka Y, Liao H, Yan SD, Stern DM, Solomon RA, Gutierrez-Ramos JC, Pinsky DJ (1996) Cerebral protection in homozygous null ICAM-1 mice after middle cerebral artery occlusion. Role of neutrophil adhesion in the pathogenesis of stroke. J Clin Invest 97:209–216. 10.1172/JCI118392 8550836 PMC507081

[B22] Daneman R, Prat A (2015) The blood-brain barrier. Cold Spring Harb Perspect Biol 7:a020412. 10.1101/cshperspect.a020412 25561720 PMC4292164

[B23] de Fougerolles AR, Qin X, Springer TA (1994) Characterization of the function of intercellular adhesion molecule (ICAM)-3 and comparison with ICAM-1 and ICAM-2 in immune responses. J Exp Med 179:619–629. 10.1084/jem.179.2.619 7905020 PMC2191386

[B24] Desai A, Park T, Barnes J, Kevala K, Chen H, Kim HY (2016) Reduced acute neuroinflammation and improved functional recovery after traumatic brain injury by α-linolenic acid supplementation in mice. J Neuroinflammation 13:253. 10.1186/s12974-016-0714-4 27663791 PMC5035510

[B125] Dietrich JB (2002) The adhesion molecule ICAM-1 and its regulation in relation with the blood-brain barrier. J Neuroimmunol 128:58–68. 12098511 10.1016/s0165-5728(02)00114-5

[B25] Ding ZM, Babensee JE, Simon SI, Lu H, Perrard JL, Bullard DC, Dai XY, Bromley SK, Dustin ML, Entman ML, et al. (1999) Relative contribution of LFA-1 and Mac-1 to neutrophil adhesion and migration. J Immunol 163:5029–5038. 10528208

[B26] Dong A, Shen J, Zeng M, Campochiaro PA (2011) Vascular cell-adhesion molecule-1 plays a central role in the proangiogenic effects of oxidative stress. Proc Natl Acad Sci USA 108:14614–14619. 10.1073/pnas.1012859108 21844360 PMC3167513

[B27] Draper K, Ponsford J (2008) Cognitive functioning ten years following traumatic brain injury and rehabilitation. Neuropsychology 22:618–625. 10.1037/0894-4105.22.5.618 18763881

[B28] Dunne JL, Ballantyne CM, Beaudet AL, Ley K (2002) Control of leukocyte rolling velocity in TNF-α-induced inflammation by LFA-1 and Mac-1. Blood 99:336–341. 10.1182/blood.V99.1.336 11756189

[B29] Dunne JL, Collins RG, Beaudet AL, Ballantyne CM, Ley K (2003) Mac-1, but not LFA-1, uses intercellular adhesion molecule-1 to mediate slow leukocyte rolling in TNF-α-induced inflammation. J Immunol 171:6105–6111. 10.4049/jimmunol.171.11.6105 14634125

[B30] Dustin ML, Staunton DE, Springer TA (1988) Supergene families meet in the immune system. Immunol Today 9:213–215. 10.1016/0167-5699(88)91216-9 3076418

[B31] Eaton K, Sallee FR, Sah R (2007) Relevance of neuropeptide Y (NPY) in psychiatry. Curr Top Med Chem 7:1645–1659. 10.2174/156802607782341037 17979774

[B32] Frank PG, Lisanti MP (2008) ICAM-1: role in inflammation and in the regulation of vascular permeability. Am J Physiol Heart Circ Physiol 295:H926–H927. 10.1152/ajpheart.00779.2008 18689494 PMC2544488

[B33] Geddes-Klein DM, Schiffman KB, Meaney DF (2006) Mechanisms and consequences of neuronal stretch injury in vitro differ with the model of trauma. J Neurotrauma 23:193–204. 10.1089/neu.2006.23.193 16503803

[B34] Geyer MA, Krebs-Thomson K, Braff DL, Swerdlow NR (2001) Pharmacological studies of prepulse inhibition models of sensorimotor gating deficits in schizophrenia: a decade in review. Psychopharmacology (Berl) 156:117–154. 10.1007/s002130100811 11549216

[B35] Goebel MU, Mills PJ (2000) Acute psychological stress and exercise and changes in peripheral leukocyte adhesion molecule expression and density. Psychosom Med 62:664–670. 11020096 10.1097/00006842-200009000-00010

[B36] González H, Pacheco R (2014) T-cell-mediated regulation of neuroinflammation involved in neurodegenerative diseases. J Neuroinflammation 11:201. 10.1186/s12974-014-0201-8 25441979 PMC4258012

[B37] Gray JA, McNaughton N (2000) The neuropsychology of anxiety: an enquiry into the functions of the septo-hippocampal system. Oxford: Oxford University Press.

[B38] Hamaï A, Meslin F, Benlalam H, Jalil A, Mehrpour M, Faure F, Lecluse Y, Vielh P, Avril MF, Robert C, Chouaib S (2008) ICAM-1 has a critical role in the regulation of metastatic melanoma tumor susceptibility to CTL lysis by interfering with PI3K/AKT pathway. Cancer Res 68:9854–9864. 10.1158/0008-5472.CAN-08-0719 19047166

[B39] Hamon M, Blier P (2013) Monoamine neurocircuitry in depression and strategies for new treatments. Prog Neuropsychopharmacol Biol Psychiatry 45:54–63. 10.1016/j.pnpbp.2013.04.009 23602950

[B40] Henderson RB, Lim LH, Tessier PA, Gavins FN, Mathies M, Perretti M, Hogg N (2001) The use of lymphocyte function-associated antigen (LFA)-1-deficient mice to determine the role of LFA-1, Mac-1, and alpha4 integrin in the inflammatory response of neutrophils. J Exp Med 194:219–226. 10.1084/jem.194.2.219 11457896 PMC2193453

[B42] Hogg N, Leitinger B (2001) Shape and shift changes related to the function of leukocyte integrins LFA-1 and Mac-1. J Leukoc Biol 69:893–898. 11404373

[B43] Hou X, Pei F (2015) Estradiol inhibits cytokine-induced expression of VCAM-1 and ICAM-1 in cultured human endothelial cells via AMPK/PPARα activation. Cell Biochem Biophys 72:709–717. 10.1007/s12013-015-0522-y 25627546

[B44] Kabadi SV, Hilton GD, Stoica BA, Zapple DN, Faden AI (2010) Fluid-percussion-induced traumatic brain injury model in rats. Nat Protoc 5:1552–1563. 10.1038/nprot.2010.112 20725070 PMC3753081

[B46] Ksiazek K, Mikuła-Pietrasik J, Catar R, Dworacki G, Winckiewicz M, Frydrychowicz M, Dragun D, Staniszewski R, Jörres A, Witowski J (2010) Oxidative stress-dependent increase in ICAM-1 expression promotes adhesion of colorectal and pancreatic cancers to the senescent peritoneal mesothelium. Int J Cancer 127:293–303. 10.1002/ijc.25036 19904754

[B47] Kumar A, Stoica BA, Loane DJ, Yang M, Abulwerdi G, Khan N, Kumar A, Thom SR, Faden AI (2017) Microglial-derived microparticles mediate neuroinflammation after traumatic brain injury. J Neuroinflammation 14:47. 10.1186/s12974-017-0819-4 28292310 PMC5351060

[B48] Landmesser U, Spiekermann S, Preuss C, Sorrentino S, Fischer D, Manes C, Mueller M, Drexler H (2007) Angiotensin II induces endothelial xanthine oxidase activation: role for endothelial dysfunction in patients with coronary disease. Arterioscler Thromb Vasc Biol 27:943–948. 10.1161/01.ATV.0000258415.32883.bf 17234726

[B49] Lindsberg PJ, Carpén O, Paetau A, Karjalainen-Lindsberg M-L, Kaste M (1996) Endothelial ICAM-1 expression associated with inflammatory cell response in human ischemic stroke. Circulation 94:939–945. 10.1161/01.CIR.94.5.939 8790029

[B50] Liu W, Chen Y, Meng J, Wu M, Bi F, Chang C, Li H, Zhang L (2018) Ablation of caspase-1 protects against TBI-induced pyroptosis in vitro and in vivo. J Neuroinflammation 15:48. 10.1186/s12974-018-1083-y 29458437 PMC5817788

[B52] Mannello F, Luchetti F, Falcieri E, Papa S (2005) Multiple roles of matrix metalloproteinases during apoptosis. Apoptosis 10:19–24. 10.1007/s10495-005-6058-7 15711919

[B53] McKee CA, Lukens JR (2016) Emerging roles for the immune system in traumatic brain injury. Front Immunol 7:556. 10.3389/fimmu.2016.00556 27994591 PMC5137185

[B54] Mickey BJ, Zhou Z, Heitzeg MM, Heinz E, Hodgkinson CA, Hsu DT, Langenecker SA, Love TM, Peciña M, Shafir T, Stohler CS, Goldman D, Zubieta JK (2011) Emotion processing, major depression, and functional genetic variation of neuropeptide Y. Arch Gen Psychiatry 68:158–166. 10.1001/archgenpsychiatry.2010.197 21300944 PMC3091621

[B55] Miller AH, Maletic V, Raison CL (2009) Inflammation and its discontents: the role of cytokines in the pathophysiology of major depression. Biol Psychiatry 65:732–741. 10.1016/j.biopsych.2008.11.029 19150053 PMC2680424

[B56] Morgane PJ, Galler JR, Mokler DJ (2005) A review of systems and networks of the limbic forebrain/limbic midbrain. Prog Neurobiol 75:143–160. 10.1016/j.pneurobio.2005.01.001 15784304

[B57] Patel RK, Prasad N, Kuwar R, Haldar D, Abdul-Muneer PM (2017) Transforming growth factor-beta 1 signaling regulates neuroinflammation and apoptosis in mild traumatic brain injury. Brain Behav Immun 64:244–258. 10.1016/j.bbi.2017.04.012 28433746

[B58] Rancan M, Otto VI, Hans VH, Gerlach I, Jork R, Trentz O, Kossmann T, Morganti-Kossmann MC (2001) Upregulation of ICAM-1 and MCP-1 but not of MIP-2 and sensorimotor deficit in response to traumatic axonal injury in rats. J Neurosci Res 63:438–446. 10.1002/1097-4547(20010301)63:5<438::AID-JNR1039>3.0.CO;2-P 11223919

[B59] Romanova LY, Mushinski JF (2011) Central role of paxillin phosphorylation in regulation of LFA-1 integrins activity and lymphocyte migration. Cell Adh Migr 5:457–462. 10.4161/cam.5.6.18219 22274710 PMC3277778

[B60] Salvador E, Neuhaus W, Foerster C (2013) Stretch in brain microvascular endothelial cells (cEND) as an in vitro traumatic brain injury model of the blood brain barrier. J Vis Exp (80):e50928. 10.3791/50928 PMC3964201 24193450

[B61] Soares HD, Hicks RR, Smith D, McIntosh TK (1995) Inflammatory leukocytic recruitment and diffuse neuronal degeneration are separate pathological processes resulting from traumatic brain injury. J Neurosci 15:8223–8233. 10.1523/JNEUROSCI.15-12-08223.1995 8613756 PMC6577921

[B62] Sobel RA, Mitchell ME, Fondren G (1990) Intercellular adhesion molecule-1 (ICAM-1) in cellular immune reactions in the human central nervous system. Am J Pathol 136:1309–1316. 1972610 PMC1877574

[B63] Springer TA (1990) Adhesion receptors of the immune system. Nature 346:425–434. 10.1038/346425a0 1974032

[B64] Stein DG, Geddes RI, Sribnick EA (2015) Recent developments in clinical trials for the treatment of traumatic brain injury. Handb Clin Neurol 127:433–451. 10.1016/B978-0-444-52892-6.00028-3 25702233

[B65] Sumagin R, Kuebel JM, Sarelius IH (2011) Leukocyte rolling and adhesion both contribute to regulation of microvascular permeability to albumin via ligation of ICAM-1. Am J Physiol Cell Physiol 301:C804–813. 10.1152/ajpcell.00135.2011 21653902 PMC3191564

[B66] Trentini A, Manfrinato MC, Castellazzi M, Tamborino C, Roversi G, Volta CA, Baldi E, Tola MR, Granieri E, Dallocchio F, Bellini T, Fainardi E, Granieri E, Castellazzi M, Casetta I, Tola MR, Fainardi E, Dallocchio F, Bellini T, Rizzo R, et al. (2015) TIMP-1 resistant matrix metalloproteinase-9 is the predominant serum active isoform associated with MRI activity in patients with multiple sclerosis. Mult Scler 21:1121–1130. 10.1177/1352458514560925 25662349

[B201] Villapol S, Balarezo MG, Affram K, Saavedra JM, Symes AJ (2015) Neurorestoration after traumatic brain injury through angiotensin II receptor blockage. Brain 138:3299–3315. 26115674 10.1093/brain/awv172 PMC4731413

[B68] Weaver LC, Bao F, Dekaban GA, Hryciw T, Shultz SR, Cain DP, Brown A (2015) CD11d integrin blockade reduces the systemic inflammatory response syndrome after traumatic brain injury in rats. Exp Neurol 271:409–422. 10.1016/j.expneurol.2015.07.003 26169930 PMC4854624

[B71] Wohleb ES, Fenn AM, Pacenta AM, Powell ND, Sheridan JF, Godbout JP (2012) Peripheral innate immune challenge exaggerated microglia activation, increased the number of inflammatory CNS macrophages, and prolonged social withdrawal in socially defeated mice. Psychoneuroendocrinology 37:1491–1505. 10.1016/j.psyneuen.2012.02.003 22386198 PMC3368999

[B72] Wurm F, Keiner S, Kunze A, Witte OW, Redecker C (2007) Effects of skilled forelimb training on hippocampal neurogenesis and spatial learning after focal cortical infarcts in the adult rat brain. Stroke 38:2833–2840. 10.1161/STROKEAHA.107.485524 17717315

[B73] Xing G, Barry ES, Benford B, Grunberg NE, Li H, Watson WD, Sharma P (2013) Impact of repeated stress on traumatic brain injury-induced mitochondrial electron transport chain expression and behavioral responses in rats. Front Neurol 4:196. 10.3389/fneur.2013.00196 24376434 PMC3859919

[B74] Yang L, Froio RM, Sciuto TE, Dvorak AM, Alon R, Luscinskas FW (2005) ICAM-1 regulates neutrophil adhesion and transcellular migration of TNF-alpha-activated vascular endothelium under flow. Blood 106:584–592. 10.1182/blood-2004-12-4942 15811956 PMC1635241

[B75] Zhang Q, Zhang J, Yan Y, Zhang P, Zhang W, Xia R (2017) Proinflammatory cytokines correlate with early exercise attenuating anxiety-like behavior after cerebral ischemia. Brain Behav 7:e00854. 10.1002/brb3.854 29201553 PMC5698870

